# Synthetic Sinapates: Antifungal, Molecular Docking, and ADMET Studies

**DOI:** 10.1002/cbdv.71569

**Published:** 2026-08-09

**Authors:** Fernando Emanuel de Sousa Ferreira, Jeremias Justo Emídio, Ézio Raul Alves de Sá, Rômulo Oliveira Barros, Paula Lima Nogueira, Ricardo Martins Ramos, Ricardo Dias de Castro, Damião Pergentino de Sousa

**Affiliations:** ^1^ Laboratory of Pharmaceutical Chemistry, Department of Pharmaceutical Sciences Federal University of Paraíba João Pessoa Paraíba Brazil; ^2^ LaPeSI–Information Systems Research Laboratory, Department of Information, Environment, Health and Food Production Federal Institute of Piauí Teresina, Piauí Brazil; ^3^ Laboratory of Experimental Pharmacology and Cell Culture, Department of Clinical and Social Dentistry Federal University of Paraiba João Pessoa Paraíba Brazil; ^4^ LaBME–Laboratory of Molecular Biology and Epidemiology, Federal Institute of Education Science and Technology of Piauí–Campus Teresina Central Teresina, Piauí Brazil

**Keywords:** Candida, chemoinformatics, fungicides, natural products, sinapic acid

## Abstract

The increasing incidence of diseases resulting from fungal infections is due to the high morbidity and mortality associated with these microorganisms. Sinapic acid is a plant‐derived phenolic compound with antifungal activity. Therefore, in this study, a collection of eleven sinapates (**1**‐**11**) was prepared and the antifungal activity of these compounds was evaluated against *Candida albicans* and *Candida tropicalis*. Compounds **4**, **5**, and **8** showed good antifungal activity. Compound **4** presented minimum inhibitory concentration (MIC) and minimum fungicidal concentration (MFC) values of 62.5 µg/mL against *C. albicans* and 15.62 µg/mL against *C. tropicalis*. Compound **5** showed MIC and MFC values of 7.81 µg/mL against both strains, while compound **8** presented MIC and MFC values of 15.62 µg/mL against *C. albicans* and 62.5 µg/mL against *C. tropicalis*. The ratio MFC/MIC<4 showed that the compounds exerted a fungicidal effect. The ergosterol assay suggests that the mechanism of action occurs via the plasma membrane. The silico study, including ADMET predictions, suggested that most of the compounds have satisfactory pharmacokinetic and toxicological properties. Therefore, sinapates appear to be promising prototypes for the development of new anti‐*Candida* drug candidates.

## Graphical abstract

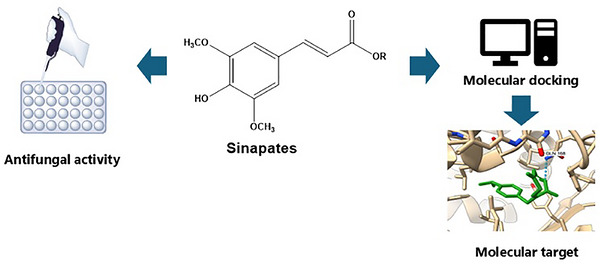

## 1 Introduction

Diseases caused by fungal infections are a current health problem, particularly in developing countries. Their incidence has increased significantly in recent decades, leading to higher morbidity and mortality [1, 2]. Among the most prevalent fungal infections, candidiasis has a high frequency of occurrence. It is a disease caused by fungi of the genus *Candida*, such as *C. albicans*. Candidiasis can manifest in localized or disseminated forms, affecting multiple organs and systemic circulation [3]. In general, these infections are easy to treat, however, in some cases, the treatment becomes ineffective, particularly in immunosuppressed patients, in whom the infection can become severe and lead to death [2, 3]. In addition, in recent decades, there has been an increase in resistance to antifungal therapy, so it is increasingly difficult to fight infections from pathogenic fungi. For this reason, the development of new antifungal agents that are less toxic and more effective is sought [4, 5]. In this context, natural products play an important role in the search for new drugs since they have various biological activities. Sinapic acid is a hydroxycinnamic acid found in nature [6, 7, 8]. Studies show that sinapic acid and its derivatives have varied pharmacological activities, including anti‐inflammatory, antioxidant, and antimicrobial actions [8, 9]. However, despite these promising biological effects, optimizing pharmacological actions through rational planning in the search for drug candidates is necessary. In this regard, obtaining derivatives of sinapic acid via structural modifications, such as changes in side chains, may influence antifungal activity against *Candida* species. Furthermore, structural modifications that alter chemical parameters, such as lipophilicity and hydrophobic interactions to enhance binding to the biological target, especially interactions with fungal membrane components, remain insufficiently understood. In this sense, the chemical modification of sinapic acid through esterification represents a useful strategy to modulate physicochemical properties, which can increase antifungal activity.

Thus, the present work aims to prepare a collection of esters derived from sinapic acid and evaluate the antifungal activity against *C. albicans* and *C. tropicalis* strains.

## 2 Results and Discussion

### 2.1 Chemistry

**SCHEME 1:**
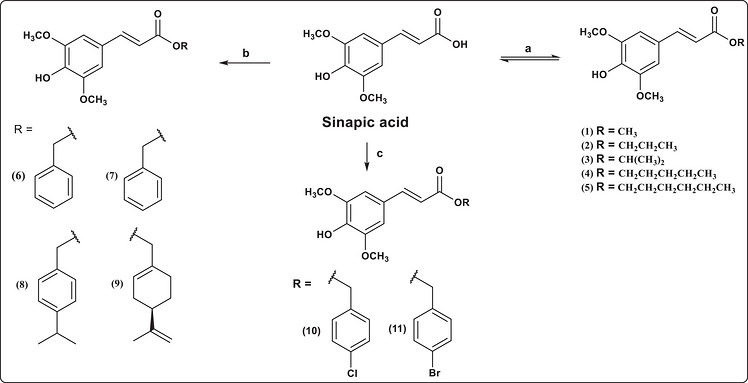
Preparation of sinapic acid derivatives (**1‐11**); (a) ROH, H_2_SO_4_, reflux; (b) ROH, DCC, DMAP, CH_2_Cl_2_; (c) Et_3_N, benzyl halide, acetone, reflux.

In the present study, eleven sinapic acid derivatives were prepared: methyl sinapate (**1**), propyl sinapate (**2**), isopropyl sinapate (**3**), pentyl sinapate (**4**), hexyl sinapate (**5**), benzyl sinapate (**6**), 4‐methylbenzyl sinapate (**7**), 4‐isopropylbenzyl sinapate (**8**), perilla sinapate (**9**), 4‐chlorobenzyl sinapate (**10**), and 4‐bromobenzyl sinapate (**11**). The sinapates were prepared using three methodologies: **1**‐**5**: prepared via Fischer Esterification, **6**‐**9**: Steglich Reaction, and **10**‐**11**: obtained through esterification with halides, as illustrated in Scheme 1.

The chemical structure of all compounds was confirmed by infrared (IR) spectroscopy and nuclear magnetic resonance of hydrogen (^1^H NMR) and carbon thirteen (^13^C NMR). Spectral data show that the compounds have satisfactory purity, above 95%. High‐resolution mass spectrometry analysis of new compounds (**5**, **7**‐**11**) was also performed. Therefore, the obtained compounds showed spectroscopic data consistent with the formation of sinapate esters. In the IR spectra, the characteristic ester carbonyl band was observed at 1670–1705 cm^−^^1^, together with bands attributed to aromatic C═C (1600 and 1510 cm^−^^1^) and C─O stretching (1100–1300 cm^−^^1^). In the ^1^H NMR spectra, doublets corresponding to the *trans* olefinic system were observed at *δ* 7.5–7.6 and 6.2–6.4 ppm (*J =* 15.9 Hz), along with a singlet at δ 3.8–3.9 ppm (6H) assigned to the methoxy groups and a singlet at *δ* 6.7–6.8 ppm corresponding to two equivalent aromatic protons of a 1,3,4,5‐tetrasubstituted ring. In the ^1^ ^3^C NMR spectra, the signal of the carbonyl carbon (167 ppm), methoxy carbons (55–57 ppm), and aromatic and olefinic carbons (100–150 ppm), including those bonded to methoxy groups (145–148 ppm), were observed. For the novel compounds, HRMS confirmed the presence of the protonated molecular ion [M+H]^+^, with m/z values consistent with the calculated values, confirming the proposed structures. The spectra and other information regarding the chemical structure of the compounds are available in the Supporting Information.

It is noteworthy that some derivatives, particularly those bearing benzyl substituents (e.g., compound **7**), were obtained in moderate to low yields (22,1%). This behavior may be attributed to steric hindrance affecting esterification efficiency, as well as side reactions associated with the Steglich protocol, including the formation of dicyclohexylurea (DCU) by‐products. In addition, partial losses during purification by column chromatography may have contributed to the reduced isolated yields. Variations in reaction time and reagent ratios were evaluated; however, no significant improvement in yield was observed, suggesting that these limitations are inherent to the reactivity of the substrates employed [10, 11]. Alternative ester preparation methods, such as the Mitsunobu reaction and the Schotten‐Baumann reaction, can be used to improve synthesis yields [12, 13]

### 2.2 Biological Activity

All compounds were pharmacologically active at the concentrations tested, however, compounds **4**, **5**, and **8** were more potent. When observing the results of the antifungal activity of the compounds against *the Candida* spp. strains, it was verified that the hexyl sinapate (**5**) emerged as the most potent compound in the series, exhibiting very strong antifungal activity, with minimum inhibitory concentration (MIC) and minimum fungicidal concentration (MFC) values of 25.33 µM against both *C. albicans* and *C. tropicalis*. These results demonstrated superior antifungal activity compared to the other derivatives and confirmed its fungicidal profile (MFC/MIC = 1). The isopropylbenzyl sinapate (**8**) showed strong bioactivity against *C. albicans* strains, with MIC and MFC of 43.83 µM, and moderate bioactivity against C. tropicalis strains, with MIC and MFC of 175.37 µM. In comparison, the pentyl sinapate (**4**) showed moderate bioactivity against *C. albicans*, with MIC and MFC equal to 213.82 µM, and strong bioactivity against *C. tropicalis* strains, with MIC and MFC equal to 53.43 µM. All compounds showed fungicidal activity (MFC/MIC ˂ 4), as shown in Table 1.

**TABLE 1:** Results of the evaluation of MIC and MFC, as well as the ratio MIC/MFC of sinapates, nystatin, and caspofungin against *Candida* spp. MIC and MFC values expressed in µg/mL (µM).

Compounds	*C. albicans* ATCC 60193			*C. tropicalis* ATCC 750		
	MIC	MFC	MFC/ MIC	MIC	MFC	MFC/ MIC
**1**	500.0 (2116.85)	500.0 (2116.85)	1	62.5 (264.60)	62.5 (264.60)	1
**2**	125.0 (473.04)	125.0 (473.04)	1	31.25 (118.26)	31.25 (118.26)	1
**3**	500.0 (1892.16)	500.0 (1892.16)	1	125.0 (473.04)	125.0 (473.04)	1
**4**	62.5 (213.82)	62.5 (213.82)	1	15.62 (53.43)	15.62 (53.43)	1
**5**	7.81 (25.33)	7.81 (25.33)	1	7.81 (25.33)	7.81 (25.33)	1
**6**	125.0 (397.70)	125.0 (397.70)	1	15.62 (49.69)	15.62 (49.69)	1
**7**	500.0 (1522.88)	500.0 (1522.88)	1	250.0 (761.44)	250.0 (761.44)	1
**8**	15.62 (43.83)	15.62 (43.83)	1	62.5 (175.37)	62.5 (175.37)	1
**9**	125.0 (348.78)	125.0 (348.78)	1	125.0 (348.78)	125.0 (348.78)	1
**10**	125.0 (343.61)	125.0 (343.61)	1	125.0 (343.61)	125.0 (343.61)	1
**11**	62.5 (167.03)	62.5 (167.03)	1	125.0 (334.06)	125.0 (334.06)	1
**Nystatin**	1.5 (1.61)	1.5 (1.61)	1	0.75 (0.80)	0.75 (0.80)	1
**Caspofungin**	0.125 (0.23)	0.25 (0.47)	2	0.125 (0.23)	0.25 (0.47)	2

The results are expressed as the mode of the findings obtained from triplicate trials conducted on three independent experiments. It was observed that the presence of a six‐carbon side chain in compound **5** resulted in a reduction in MIC and MFC when compared to compound **4** with a five‐carbon side chain. The six‐carbon side chain of compound **5** also showed better activity when compared to compound **8**, which has a benzyl ring with an isopropyl branching at the *para‐*position.

Regarding the antifungal potency of the short‐chain compounds (**1**, **2**, and **3**), it is noted that there was no significant reduction in MIC and MFC as the number of carbons in the side chain increased from one to three. In this way, compound **1** exhibited MIC values of 2116.85 µM against *C. albicans* and 264.6 µM against *C. tropicalis*. Compound **2** showed MIC values of 125.0 and 118.26 µM against *C. albicans* and *C. tropicalis*, respectively. In contrast, compound **3** presented MIC values of 1892.16 and 473.04 µM against *C. albicans* and *C. tropicalis*, respectively. However, compounds **4**, with a pentyl substituent, and **5**, more lipophilic due to the longest carbon chain, showed considerably lower MIC and MFC compared to the two strains tested. In numerical terms, compound **4** exhibited MIC and MFC values of 213.82 µM against *C. albicans* and 53.43 µM against *C. tropicalis*, while compound **5** showed MIC and MFC values of 25.33 µM against both strains. Thus, the presence of open chains with five and six carbons is relevant for bioactivity. Therefore, it was observed that the increase in the nonpolar character enhances the activity of the compounds. In the structure‐activity relationship analysis, MIC values ​​were expressed in µM for a proper comparison of the influence of lipophilicity and structural characteristics of the compounds. An increase in lipophilicity, associated with the length of the alkyl side chain, was monitored by an improvement in antifungal activity. This trend was observed when comparing short‐chain derivatives (compounds **1**–**3**) with medium‐chain derivatives (compounds **4** and **5**), which showed lower MIC values ​​against both *Candida* strains.

Compounds with higher molecular weight, particularly those bearing aromatic substituents (compounds **6**–**11**), did not show a proportional increase in activity and, in several cases, displayed reduced potency compared to compound **5**. The data suggest that the potentiation of antifungal activity is associated with alterations in lipophilic character related to the side chain structure, although this effect is not uniform across all derivatives. This behavior indicates that increased lipophilicity in sinapate derivatives may enhance interaction with fungal cell membranes, thus increasing antifungal activity. Regarding the volume and molecular weight of the compounds, no correlation was observed with the results of bioactivity.

The structure–activity relationship observed for the sinapate derivatives was consistent with previous reports on phenolic compounds and hydroxycinnamic acid esters. The increase in antifungal activity associated with longer alkyl chains, particularly in compound **5**, suggested that lipophilicity played a crucial role in enhancing membrane interaction, as previously described for phenolic esters that disrupt fungal cell membranes by increasing permeability and promoting leakage of intracellular components [14, 15]. This interpretation was further supported by studies demonstrating that hydroxycinnamic derivatives exhibit improved antifungal activity when their hydrophobic character is increased, facilitating interaction with ergosterol‐rich membranes [16]. In contrast, the reduced activity observed for benzyl‐substituted derivatives, despite their favorable docking results, suggested that steric effects and molecular orientation may limit their effective interaction with the lipid bilayer, a behavior also reported for bulky phenolic derivatives [17]. Furthermore, the absence of a direct correlation between molecular weight and antifungal activity reinforced that lipophilicity, rather than molecular size, was the dominant factor in the biological activity, in agreement with previous structure–activity relationship studies on cinnamic acid derivatives [18]. Taken together, these findings supported that membrane disruption was the primary mechanism of action, which was consistent with the results obtained in the ergosterol assay.

When comparing compound **5** with compounds **6** and **7**, which have aryl substituents, a reduction in MIC and MFC values was observed. Compounds **6** and **7** presented MIC values of 397.70 and 1522.88 µM against *C. albicans*, and 49.69 and 761.44 µM against *C. tropicalis*, respectively. The addition of an aryl substituent with an isopropyl chain at the *para*‐position in compound **8** resulted in an increase in antifungal activity against *C. albicans* strains compared to compounds **6** and **7**, with an MIC of 43.83 µM. Regarding *C. tropicalis*, the same pattern was observed for compound **7**, with MIC of 761.44 µM; however, the MIC and MFC values of compound **6** were lower than those of compound **8** against *C. tropicalis*, with values of 49.69 and 175.37 µM, respectively. This behavior can be explained by variations in the chemical structure of the substituent groups, as compound **6** lacks a substituent at the *para*‐position, whereas compound **7** has a methyl group at this position on the benzyl ring. Comparing compounds **8** and **9**, it is noted that the latter showed a reduction in antifungal potency for both studied species compared to compound **8**, with MIC of 348.78 µM. This can be attributed to the replacement of the 4‐isopropylbenzyl group in compound **8** with the perilla substituent, a cyclic terpenoid, in compound **9**. On the other hand, compounds **10** and **11**, which have halogen substituents (chlorine and bromine, respectively) at the *para*‐position of the benzyl ring, exhibited weak antifungal activity, with MIC values of 343.61 µM for both *C. albicans* and *C. tropicalis* (compound **10**), and 167.03 µM against *C. albicans* and 334.06 µM against *C. tropicalis* (compound **11**).

Perez‐Castillo et al. (2020) evaluated the antifungal potential of methyl sinapate against *C. albicans* strains (ATCC 10231), obtaining MIC values of 931 µM, a result like those obtained in the present study [17]. Lima et al. (2017) showed that the insertion of a hydroxyl group in the benzene ring improves antifungal activity, and that the addition of a second hydroxyl group further enhances biological activity. However, the insertion of a third hydroxyl group led to a reduction in activity [19].

Therefore, the hydroxyl group present in the study molecules interferes with the antifungal action [19]. Thus, these studies confirm that both the type of substituents and their position on the aromatic ring play a crucial role in modulating antifungal activity and are consistent with the structure‐activity relationships observed in the present work. In fact, the therapeutic potential of this chemical class is not limited to antimicrobial activity. These types of compounds also possess various pharmacological properties, such as antitumor, antidepressant, antidiabetic, and anti‐inflammatory effects [20]. Therefore, further research should be conducted to explore their application in various organic disorders associated with fungal infections.

From the results obtained, an analysis was carried out to identify the mechanism of action of the compounds, considering that the collection of prepared sinapates is structurally related, changing only the side chain and maintaining the sinapic structure in all compounds. Compound **4** showed moderate bioactivity and compounds **5** and **8** displayed strong bioactivity against *C. albicans* ATCC 60193 and *C. tropicalis* ATCC 750 strains.

Then, the mechanism of action of the most pharmacologically potent compounds (**4**, **5**, and **8**) was evaluated by means of sorbitol and ergosterol assays, using the *C. albicans* ATCC 60193 strain. The analysis of the results (Table 2) shows that the MIC of the compounds increased in the presence of exogenous ergosterol. In more discriminated results, the MIC of compounds **4**, **5**, and **8** in the absence of exogenous ergosterol was, respectively, in the value of 213.8, 25.3, and 43.8 µM, whereas in the presence of exogenous ergosterol, the respective MIC values increased to 3421.2, 3421.2, and 1403 µM.

**TABLE 2:** MIC values of **4**, **5**, and **8** and nystatin in the absence and presence of exogenous ergosterol (1.008 mM) against *C. albicans* strain ATCC 60193. Values expressed in µg/mL (µM).

*C. albicans* ATCC 60193											
4			5			8			Nystatin		
Concentration µg/mL	Absence of ergosterol	Presence of ergosterol	Concentration µg/mL	Absence of ergosterol	Presence of ergosterol	Concentration µg/mL	Absence of ergosterol	Presence of ergosterol	Concentration µg/mL	Absence of ergosterol	Presence of ergosterol
1000 (3421.2)	—	—	1000 (3243.2)	—	—	1000 (2806.1)	—	—	48 (51.8)	—	—
500 (1710.6)	—	+	500 (1621.6)	—	+	500 (1403)	—	—	24 (25.9)	—	+
250 (855.3)	—	+	250 (810.8)	—	+	250 (701.5)	—	+	12 (12.95)	—	+
125 (427.6)	—	+	125 (405.4)	—	+	125 (350.7)	—	+	6.0 (6.4)	—	+
62.5 (213.8)	—	+	62.5 (202.7)	—	+	62.5 (175.3)	—	+	3.0 (3.2)	—	+
31.25 (106.9)	+	+	31.25 (101.3)	—	+	31.25 (87.6)	—	+	1.5 (1.6)	—	+
15.62 (43.8)	+	+	15.62 (50.6)	—	+	15.62 (43.8)	—	+	0.75 (0.8)	+	+
7.81 (26.7)	+	+	7.81 (25.3)	—	+	7.81 (21.9)	+	+	0.37 (0.39)	+	+

Observations: +, fungal growth; ‐, absence of fungal growth.

In view of the results of the ergosterol assay, it was inferred that the compounds have a mechanism of action via the plasma membrane of the fungal cell, considering that the substances that act on the ergosterol of the membrane tend to bind to the exogenous ergosterol present in the culture medium. In this sense, the results of nystatin worked as predicted, since the MIC value in the absence of exogenous ergosterol was 1.6 µM, while in the presence of exogenous ergosterol, the MIC increased to 51.8 µM, thus confirming its mechanism of action via the plasma membrane of the fungal cell. These findings agreed with the results found by Melo et al. (2021) in which it was evidenced through the ergosterol assay, using the 4‐chlorobenzyl *p*‐coumarate against strains of *C. albicans*, that phenolic compounds act on the plasma membrane of *C. albicans* [21]. This mechanism is widely reported for phenolic compounds, which are known to interact with membrane sterols, leading to increased permeability and cell death.

To evaluate the mechanism of action via the cell wall, the sorbitol assay was performed, however, it was observed that the MIC results of the tested compounds (Table 3) did not change in the absence and presence of sorbitol, therefore, they remained with values of 213.8, 25.3, and 43.8 µM for the compounds **4**, **5,** and **8,** respectively. Therefore, the compounds have no action on the fungal cell wall. This result agrees with previous studies indicating that hydroxycinnamic derivatives preferentially target the fungal cell membrane rather than the cell wall. On the other hand, the result of the positive control, caspofungin, worked as expected, so the MIC value in the absence of sorbitol was 0.11 µM and increased significantly when it was in contact with sorbitol, thus presenting a value of 1.80 µM. Therefore, its mechanism of action via the fungal cell wall is ratified.

**TABLE 3:** MIC values of **4**, **5**, and **8** and caspofungin in the absence and presence of sorbitol (0.8 M) against *C. albicans* strain ATCC 60193. Values expressed in µg/mL (µM).

*C. albicans* ATCC 60193											
4			5				8		Caspofungin		
Concentration µg/mL	Absence of sorbitol	Presence of sorbitol	Concentration µg/mL	Absence of sorbitol	Presence of sorbitol	Concentration µg/mL	Absence of sorbitol	Presence of sorbitol	Concentration µg/mL	Absence of sorbitol	Presence of sorbitol
1000 (3421.2)	—	—	1000 (3243.2)	—	—	1000 (2806.1)	—	—	4 (3,60)	—	—
500 (1710.2)	—	—	500 (1621.6)	—	—	500 (1403)	—	—	2 (1.80)	—	—
250 (855.3)	—	—	250 (810.8)	—	—	250 (701.5)	—	—	1 (0.90)	—	+
125 (427.6)	—	—	125 (405.4)	—	—	125 (350.7)	—	—	0.50 (0,45)	—	+
62.5 (213.8)	—	—	62.5 (202.7)	—	—	62.5 (175.3)	—	—	0.25 (0.22)	—	+
31.25 (106.9)	+	+	31.25 (101.3)	—	—	31.25 (87.6)	—	—	0.125 (0.11)	—	+
15.62 (43.8)	+	+	15.62 (50.6)	—	—	15.62 (43.8)	—	—	0.062 (0.056)	+	+
7.81 (26.7)	+	+	7.81 (25.3)	—	—	7.81 (21.9)	+	+	0.031 (0.028)	+	+

Observations: +, fungal growth; ‐, absence of fungal growth.

### 2.3 In Silico Analysis

The in silico study was carried out with the objective of identifying possible toxic effects of the sinapates. In this context, the objective of the research was to evaluate parameters related to carcinogenesis, hepatotoxicity, immunotoxicity, and cytotoxicity. LD_50_, pharmacokinetic, and solubility parameters were also analyzed.

### 2.4 ADMET Predictions Results

Tables 4 and 5 present the results of ADMET predictions for the eleven compounds. It is noteworthy to highlight that the compounds presented pharmacokinetic parameter results, according to an in silico prediction study, which are important for molecular docking and subsequent analyses for validation.

**TABLE 4:** Toxicity parameters by In Silico Toxicology and ProToxII.

Parameters	1	2	3	4
Toxicity				
Mutagenicity (Salmonella typhimurium)	Not mutagenic	Not mutagenic	Not mutagenic	Not mutagenic
Carcinogenicity in rats	Not carcinogenic	Not carcinogenic	Not carcinogenic	Not carcinogenic
Carcinogenicity in rodents	Not carcinogenic	Not carcinogenic	Not carcinogenic	Not carcinogenic
Maximum Recommended Daily Dose (Human) (mg/kg_weight/day)	2.67	2.54	2.25	10.8
Hepatotoxicity	Active	Inactive	Active	Inactive
Immunotoxicity	Active	Active	Active	Active
Cytotoxicity	Inactive	Inactive	Inactive	Inactive
Predicted LD50	978 mg/kg	978 mg/kg	978 mg/kg	9,600 mg/kg

Parameter	5	6	7	8
Toxicity				
Mutagenicity (*Salmonella typhimurium*)	Non‐mutagenic	Non‐mutagenic	Non‐mutagenic	Non‐mutagenic
Carcinogenicity in rats	Non‐carcinogenic	Non‐carcinogenic	Non‐carcinogenic	Non‐carcinogenic
Carcinogenicity in rodents	Non‐carcinogenic	Non‐carcinogenic	Non‐carcinogenic	Non‐carcinogenic
Maximum Recommended Daily Dose (Human) (mg/kg_weight/day)	11.3	4.39	12.0	—
Hepatotoxicity	Inactive	Inactive	Inactive	Inactive
Immunotoxicity	Active	Active	Active	Active
Cytotoxicity	Inactive	Inactive	Inactive	Inactive
Predicted LD_50_	9,600 mg/kg	978 mg/kg	978 mg/kg	978 mg/kg

Parameter	9	10	11
Toxicity			
Mutagenicity (*Salmonella typhimurium*)	Non‐mutagenic	Non‐mutagenic	Non‐mutagenic
Carcinogenicity in rats	Non‐carcinogenic	Non‐carcinogenic	Non‐carcinogenic
Carcinogenicity in rodents	Non‐carcinogenic	Non‐carcinogenic	Non‐carcinogenic
Maximum Recommended Daily Dose (Human) (mg/kg_weight/day)	—	—	—
Hepatotoxicity	Inactive	Inactive	Inactive
Immunotoxicity	Active	Active	Active
Cytotoxicity	Inactive	Inactive	Inactive
Predicted LD_50_	9,600 mg/kg	978 mg/kg	978 mg/kg

**TABLE 5:** Water solubility, pharmacokinetic, and druglikeness parameters evaluated by SwissADME.

Parameters	1	2	3	4
ADME				
Blood‐brain barrier permeation	Yes	Yes	Yes	Yes
P‐glycoprotein substrate	No	No	No	No
Human intestinal absorption	High	High	High	High
Water solubility	Soluble	Soluble	Soluble	Soluble
**Druglikeness**				
Lipinski's Rule of Five	Adequate	Adequate	Adequate	Adequate

Parameter	5	6	7	8
ADME				
Blood‐brain barrier permeation	Yes	Yes	Yes	Yes
*P*‐glycoprotein substrate	No	No	No	No
Human intestinal absorption	High	High	High	High
Water solubility	Soluble	Soluble	Moderately Soluble	Moderately Soluble
**Druglikeness**				
Lipinski's Rule of Five	Adequate	Adequate	Adequate	Adequate

Parameter	9	10	11
ADME			
Blood‐brain barrier permeation	Yes	Yes	Yes
P‐glycoprotein substrate	No	No	No
Human intestinal absorption	High	High	High
Water solubility	Moderately Soluble	Moderately Soluble	Moderately Soluble
**Druglikeness**			
Lipinski's Rule of Five	Adequate	Adequate	Adequate

Table 4 shows the predicted toxicity parameters for the compounds, indicating an absence of toxicity in rats and mice, as well as a lack of mutation capacity. The highest recommended daily doses are for compounds **7**, **5**, and **4**, making it impossible to predict for compounds **8**, **9**, **10**, and **11**. Only substances **1** and **3** show a high hepatotoxicity capacity; the compounds in Table 4 show a favorable immunotoxicity profile and an absence of cytotoxicity in preliminary studies. The lowest predicted LD_50_ is for compounds **4**, **5**, and **9**, with an estimated value of 9,600 mg/kg, indicating a higher mortality rate of 50% in a sample population. Thus, the silico results for toxicity corroborate the data observed in the experimental study, demonstrating the potential of these substances.

Table 5 shows the predicted ADME parameters for the compounds, demonstrating that all obey Lipinski's rule of five, an important rule for predicting the oral bioavailability of compounds for the discovery of new drugs. Furthermore, they exhibit high intestinal absorption, aiding in the oral absorption of these substances by the human body. Compounds **1** to **6** are completely soluble in water, while compounds **7** to **11** have moderate solubility. None of the compounds act as *P*‐glycoprotein substrates; therefore, they will not be transported out of cells via efflux pumps using this protein, allowing for molecular concentration and intracellular absorption.

The compounds investigated in Table 5 show great potential in crossing the blood‐brain barrier, an important natural barrier between the blood and the central nervous system, but which the vast majority of substances fail to cross, hindering the treatment of some diseases. However, the low ability of drugs to enter the brain leads to low therapeutic efficacy in the treatment of neurodegenerative diseases and brain cancer, worsening side effects due to accumulation in other organs and tissues of the body [22, 23, 24]. Therefore, these compounds present good preliminary results for analysis and the development of experimental studies.

### 2.5 Results of Molecular Docking

All the proteins selected for the study are present in biosynthetic pathways essential for cell survival, especially in the production of structural components of the cell, as well as cell replication and growth. Squalene Monooxygenase, Lanosterol 14‐alpha‐demethylase, and Delta(14)‐sterol reductase participate in the cholesterol pathway in humans and the ergosterol pathway in fungi [25, 26]. 1,3‐beta‐glucan synthase participates in the synthesis for the formation of the fungal cell wall [26]. Thymidylate synthase is essential for DNA replication and cell division [27]. Thus, they have been considered classic targets for drug design and development, since their inhibition directly compromises cell survival.

The results of the molecular docking experiments are presented in Table 6. Comparative analysis of binding affinity in the five enzymatic targets with the chemical structures of sinapates reveals that the presence of benzyl substituents in the benzyl sinapate (**6**), 4‐methylbenzyl sinapate (**7**), 4‐isopropylbenzyl sinapate (**8**), 4‐chlorobenzyl sinapate (**10**), and 4‐bromobenzyl sinapate (**11**) result in superior binding stabilities compared to the alkyl‐series (methyl to hexyl sinapates). This trend suggests that the presence of an aromatic moiety in the ester side chain facilitates enhanced lipophilic interactions and potential π‐stacking within the hydrophobic pockets of the receptors.

**TABLE 6:** Results of molecular docking simulations with squalene monooxygenase and various ligands.

Proteins	Ligands	ΔGbind (kcal/mol)^a^	Amino acids interacting through hydrogen bonds^b^	Amino acids that make hydrophobic interactions^b^
Squalene Monooxygenase (PDB: 6C6P)	NB‐598 (Co‐crystallized Ligand. Redocking validation: RMSD (Å) 0.66)	−11.69	None	Tyr195(A), Leu509(A), Leu324(A), Leu416(A), Phe477(A), Gln168(A), Ala322(A), Leu333(A), Leu473(A)
	4‐Chlorobenzyl Sinapate (**10**)	−9.69	Gln168(A) [5.75 Å]	Tyr195(A), Phe166(A), Leu416(A), Leu333(A), Leu324(A), Leu473(A), Phe477(A), Leu509(A), Tyr335(A), Thr417(A)
	Tomudex	−9.58	Tyr195(A) [7.98 Å], Glu323(A) [7.98 Å], Ile334(A) [6.24 Å]	Leu333(A), Leu324(A), Phe495(A), Pro505(A), Tyr335(A), Leu416(A), Leu509(A), Phe166(A), Ile208(A), Thr417(A), Leu473(A), Ala322(A), Gly418(A), Phe477(A), Ile197(A), Leu469(A), Val502(A), Val506(A)
	4‐Bromobenzyl Sinapate (**11**)	−9.57	Gln168(A) [5.78 Å]	Phe166(A), Tyr195(A), Leu416(A), Leu333(A), Leu473(A), Leu324(A), Tyr335(A), Phe477(A), Leu509(A), Thr417(A)
	4‐Methylbenzyl Sinapate (**7**)	−9.37	Gln168(A) [6.14 Å]	Phe166(A), Tyr195(A), Leu416(A), Leu333(A), Leu324(A), Tyr335(A), Phe477(A), Leu509(A), Ala322(A), Thr417(A), Gly418(A), Leu473(A)
	Benzyl Sinapate (**6**)	−8.83	Gln168(A) [6.05 Å]	Tyr195(A), Leu509(A), Leu416(A), Leu333(A), Phe166(A), Leu324(A), Tyr335(A), Thr417(A), Gly418(A)
	4‐Isopropylbenzyl Sinapate (**8**)	−8.29	Gln168(A) [5.03 Å]	Phe166(A), Tyr195(A), Leu324(A), Leu416(A), Leu509(A), Phe477(A), Ala322(A), Leu333(A), Thr417(A), Gly418(A), Leu473(A)
	Perilla Sinapate (**9**)	−7.81	Gln168(A) [2.92 Å], Tyr210(A) [2.92 Å]	Tyr195(A), Phe166(A), Thr417(A), Gly418(A), Leu509(A), Leu324(A), Tyr335(A), Leu416(A), Leu333(A)
	Hexyl Sinapate (**5**)	−7.67	Gln168(A) [4.96 Å], Tyr210(A) [4.96 Å]	Phe166(A), Tyr195(A), Leu324(A), Leu416(A), Thr417(A), Leu333(A), Tyr335(A), Ala322(A), Gly418(A), Leu473(A), Phe477(A), Leu509(A)
	Pentyl Sinapate (**4**)	−7.41	Gln168(A) [5.08 Å], Tyr210(A) [5.08 Å]	Phe166(A), Tyr195(A), Thr417(A), Leu416(A), Tyr335(A), Leu509(A), Leu324(A), Leu333(A), Gly418(A), Leu473(A)
	Propyl Sinapate (**2**)	−7.30	Gln168(A) [2.91 Å], Tyr210(A) [2.91 Å]	Tyr195(A), Phe166(A), Thr417(A), Gly418(A), Leu324(A), Leu333(A), Tyr335(A), Leu416(A), Leu509(A)
	Isopropyl Sinapate (**3**)	−6.88	Gln168(A) [3.07 Å], Tyr210(A) [3.07 Å]	Phe166(A), Tyr195(A), Thr417(A), Leu324(A), Leu333(A), Tyr335(A), Leu416(A), Leu509(A), Gly418(A)
	Methyl Sinapate (**1**)	−6.47	Gln168(A) [2.99 Å], Tyr210(A) [2.99 Å]	Phe166(A), Tyr195(A), Thr417(A), Tyr335(A), Leu509(A), Leu333(A), Leu416(A), Gly418(A)
	Fluconazole	−6.30	Leu324(A) [2.84 Å], Met196(A) [2.84 Å]	Tyr195(A), Leu416(A), Glu323(A), Thr417(A), Phe166(A), Leu333(A), Leu473(A), Leu509(A), Ile197(A), Phe477(A), Phe495(A), Gln168(A), Ala322(A), Ile334(A), Tyr335(A), Gly418(A)
Lanosterol 14α‐Demethylase (PDB: 4WMZ)	Tomudex	−9.75	His381(A) [3.07 Å], Met509(A) [3.07 Å], Ser382(A) [5.49 Å]	Hem601(A), Val311(A), Leu380(A), Tyr126(A), Ile139(A), Tyr140(A), Phe236(A), Gly310(A), Gly314(A), Phe384(A), Pro238(A), Leu383(A), Thr507(A)
	Perilla Sinapate (**9**)	−9.72	His381(A) [2.86 Å], Ser382(A) [2.86 Å]	Met509(A), Tyr72(A), Tyr126(A), Leu380(A), Ser508(A), Pro238(A), Leu383(A), Phe384(A)
	4‐Isopropylbenzyl Sinapate (**8**)	−9.32	Tyr126(A) [8.98 Å], Tyr140(A) [8.98 Å], Ser382(A) [8.98 Å]	Hem601(A), Val311(A), Leu380(A), Ile139(A), Gly310(A), Gly314(A), Leu383(A), Met509(A)
	4‐Methylbenzyl Sinapate (**7**)	−9.22	Tyr140(A) [3.79 Å]	Hem601(A), Tyr126(A), Val311(A), Leu380(A), Ile139(A), Phe236(A), Gly310(A), Gly314(A), Leu383(A)
	4‐Chlorobenzyl Sinapate (**10**)	−8.94	Tyr140(A) [3.69 Å]	Hem601(A), Tyr126(A), Val311(A), Ile139(A), Gly310(A), Leu380(A), Gly314(A), Leu383(A)
	4‐Bromobenzyl Sinapate (**11**)	−8.90	Tyr126(A) [7.40 Å], Tyr140(A) [7.40 Å]	Hem601(A), Leu380(A), Met509(A), Gly310(A), Val311(A), Leu383(A)
	NB‐598	−8.63	None	Hem601(A), Tyr140(A), Ile139(A), Gly310(A), Val311(A), Tyr126(A), Leu380(A)
	Benzyl Sinapate (**6**)	−8.57	Tyr140(A) [3.79 Å]	Hem601(A), Tyr126(A), Ile139(A), Gly310(A), Leu380(A), Val311(A), Gly314(A)
	Fluconazole (Co‐crystallized Ligand. Redocking validation: RMSD (Å) 1.98)	−8.09	None	Hem601(A), Phe236(A), Ile139(A), Tyr140(A), Val311(A), Gly314(A), Thr130(A), Gly310(A), Leu380(A), Leu129(A), Thr318(A), Val510(A)
	Hexyl Sinapate (**5**)	−8.03	Hem601(A) [2.33 Å], Cys470(A) [2.33 Å]	Tyr126(A), Phe134(A), Tyr140(A), Phe236(A), Gly310(A), Gly314(A), Thr130(A), Val311(A), Leu380(A), Leu383(A)
	Pentyl Sinapate (**4**)	−7.98	None	Hem601(A), Tyr126(A), Phe134(A), Tyr140(A), Gly310(A), Ile139(A), Val311(A), Gly314(A), Leu380(A), Leu383(A)
	Propyl Sinapate (**2**)	−7.48	His381(A) [2.91 Å], Ser382(A) [2.91 Å]	Met509(A), Tyr72(A), Leu380(A), Ser508(A), Tyr126(A), Pro238(A), Leu383(A), Phe384(A)
	Isopropyl Sinapate (**3**)	−7.29	None	Hem601(A), Val311(A), Gly314(A), Gly310(A), Ile139(A), Thr318(A), Leu380(A)
	Methyl Sinapate (**1**)	−7.07	His381(A) [5.86 Å], Tyr72(A) [5.86 Å], Ser382(A) [6.91 Å]	Phe384(A), Met509(A), Leu380(A), Phe506(A), Ser508(A)
Δ(14)‐Sterol Reductase (PDB: 4QUV)	Benzyl Sinapate (**6**)	−8.08	Arg395(A) [5.06 Å], Asn359(A) [5.06 Å]	Tyr245(A), Tyr241(A), Ndp501(A), Asp244(A), Ala251(A), Val252(A), Thr255(A), Phe312(A), Arg313(A), Tyr360(A)
	4‐Chlorobenzyl Sinapate (**10**)	−8.00	Arg395(A) [4.24 Å], Asn359(A) [4.24 Å]	Tyr241(A), Tyr245(A), Asp244(A), Val252(A), Phe312(A), Asn316(A), Ndp501(A), Ala251(A), Thr255(A), Arg313(A)
	Tomudex	−7.99	Thr255(A) [6.32 Å], Arg313(A) [6.32 Å]	Tyr245(A), Arg395(A), Val252(A), Asp257(A), Met206(A), Tyr241(A), His248(A), Ala251(A), Tyr279(A), Phe312(A), Leu391(A), Glu201(A), Trp256(A), Asp363(A), Arg398(A), Ndp501(A)
	NB‐598	−7.89	None	Tyr241(A), Tyr245(A), Ala251(A), Val252(A), Phe312(A), Asp244(A), His248(A), Ndp501(A)
	Perilla Sinapate (**9**)	−7.89	Arg313(A) [5.95 Å]	Tyr245(A), His248(A), Phe312(A), Ndp501(A), Tyr241(A), Asp244(A), Ala251(A), Val252(A), Asn316(A), Asp363(A), Arg395(A)
	4‐Bromobenzyl Sinapate (**11**)	−7.49	Asn359(A) [5.02 Å]	Tyr241(A), Tyr245(A), Val252(A), Phe312(A), His248(A), Asn316(A), Ndp501(A), Asp244(A), Ala251(A), Thr255(A), Arg313(A)
	4‐Methylbenzyl Sinapate (**7**)	−7.44	Asn359(A) [5.53 Å], Arg395(A) [5.53 Å]	Tyr241(A), Phe312(A), Tyr245(A), Asn316(A), Ndp501(A), Asp244(A), Val252(A), Thr255(A), Arg313(A), Tyr360(A)
	4‐Isopropylbenzyl Sinapate (**8**)	−7.38	Tyr279(A) [6.78 Å], Tyr387(A) [6.78 Å], Glu201(A) [6.78 Å]	Trp274(A), Leu275(A), Gly270(A), Leu32(A), Met206(A), Asp271(A)
	Fluconazole	−7.07	None	Trp274(A), Met266(A), Leu267(A), Gly270(A), Arg395(A), Asp257(A), Tyr387(A), Leu32(A), Trp256(A), Glu261(A), Trp269(A), Leu391(A), Arg398(A)
	Isopropyl Sinapate (**3**)	−6.35	None	Tyr241(A), Tyr245(A), Phe312(A), Ala251(A), Val252(A), Asp244(A), His248(A), Arg313(A), Asn316(A)
	Propyl Sinapate (**2**)	−6.20	Arg313(A) [6.36 Å]	Tyr245(A), Tyr241(A), Asp244(A), Val252(A), Phe312(A), Ala251(A), His248(A), Ndp501(A)
	Pentyl Sinapate (**4**)	−6.14	Arg395(A) [7.09 Å]	Tyr241(A), Tyr245(A), Phe312(A), His248(A), Ala251(A), Ndp501(A), Asp244(A), Arg313(A), Asn316(A), Asn359(A)
	Hexyl Sinapate (**5**)	−6.11	Tyr241(A) [5.20 Å], Arg395(A) [5.20 Å]	Tyr245(A), Phe312(A), Asp363(A), Asp244(A), His248(A), Ala251(A), Arg313(A)
	Methyl Sinapate (**1**)	−6.03	Arg398(A) [3.75 Å], Trp256(A) [3.75 Å]	Ndp501(A), Lys259(A), Thr255(A), His260(A), Tyr360(A), Arg395(A), Asp399(A), Met402(A)
Thymidylate Synthase (PDB: 6K7S, chain A)	Tomudex	−8.17	Tyr234(A) [9.46 Å], Asp194(A) [9.46 Å], Ser192(A) [7.66 Å], Ala288(A) [7.66 Å]	Phe201(A), Phe56(A), Ile84(A), Trp85(A), Ala193(A), Gly198(A), Leu168(A), Cys171(A), Leu197(A), His232(A), Met287(A)
	4‐Isopropylbenzyl Sinapate (**8**)	−7.99	Asn202(A) [7.84 Å], Cys171(A) [7.66 Å]	Phe201(A), Ile84(A), Trp85(A), Glu63(A), His172(A), Leu197(A), Met287(A)
	Perilla Sinapate (**9**)	−7.81	Asp194(A) [3.12 Å], Asn202(A) [3.12 Å]	Ile84(A), Gly198(A), Phe201(A), Ser192(A), Ala193(A), Cys171(A), His172(A), Gln190(A), Leu197(A)
	NB‐598	−7.62	None	Phe201(A), Glu63(A), Ile84(A), His172(A), Gly198(A), Phe67(A), Trp85(A), Tyr111(A), Leu197(A), Asn202(A)
	4‐Methylbenzyl Sinapate (**7**)	−7.51	None	Phe201(A), Ile84(A), Trp85(A), Glu63(A), Asn202(A), His172(A), Leu197(A), Gly198(A), Met287(A)
	4‐Chlorobenzyl Sinapate (**10**)	−7.41	None	Phe201(A), Ile84(A), Glu63(A), Trp85(A), Asn202(A), His172(A), Leu197(A), Gly198(A), Met287(A)
	Benzyl Sinapate (**6**)	−7.35	None	Phe201(A), Ile84(A), Glu63(A), Trp85(A), Asn202(A), Cys171(A), His172(A), Met287(A)
	4‐Bromobenzyl Sinapate (**11**)	−7.07	Asn202(A) [4.60 Å]	Phe201(A), Trp85(A), Ile84(A), Glu63(A), Cys171(A), His172(A), Leu197(A), Gly198(A), Met287(A)
	Fluconazole	−6.76	None	Phe201(A), Ile84(A), Trp85(A), Cys171(A), Asn202(A), His172(A), Asp194(A), Leu197(A), Gly198(A), Met287(A), Asn88(A), Leu168(A)
	Pentyl Sinapate (**4**)	−6.17	Asp194(A) [7.40 Å]	Phe201(A), Ile84(A), Asn202(A), Cys171(A), Ser192(A), Ala193(A), Gly198(A), Glu63(A), Trp85(A), His172(A), Gln190(A)
	Propyl Sinapate (**2**)	−6.13	Asp194(A) [2.96 Å], Asn202(A) [2.96 Å]	Phe201(A), Cys171(A), Ala193(A), Gly198(A), Ile84(A), Gln190(A)
	Hexyl Sinapate (**5**)	−5.96	Asp194(A) [4.85 Å], Asn202(A) [4.85 Å]	Trp85(A), Ile84(A), Phe201(A), Cys171(A), Ala193(A), Leu197(A), Gly198(A), Glu63(A), Gln190(A)
	Isopropyl Sinapate (**3**)	−5.86	Asp194(A) [7.76 Å]	Phe201(A), Asn202(A), Cys171(A), Gly198(A), Ile84(A), His172(A), Gln190(A), Ala193(A)
	Methyl Sinapate (**1**)	−5.77	Asp194(A) [2.97 Å], Ser192(A) [2.97 Å]	Cys171(A), Ala193(A), Asn202(A), Gln190(A), Gly198(A), His232(A)
1,3‐β‐Glucan Synthase (GSC2, AlphaFold, UniProt P40989)	Tomudex	−8.32	—	—
	Perilla Sinapate (**9**)	−7.99	Arg462(A) [2.88 Å], Ser1572(A) [2.88 Å]	Lys398(A), Gly1440(A), Asp1570(A), Ala1571(A), Ser399(A), Glu400(A), Gly1439(A)
	4‐Isopropylbenzyl Sinapate (**8**)	−7.85	Ser1434(A) [6.50 Å], Arg1551(A) [6.50 Å]	Asp1435(A), Ala1283(A), Ser1430(A), Ala1431(A), Arg1463(A), Phe1464(A), Ser1467(A), Thr1279(A), Ile1427(A), Gly1440(A), Ala1441(A)
	4‐Methylbenzyl Sinapate (**7**)	−7.43	Ser1434(A) [6.56 Å], Arg1551(A) [6.56 Å]	Asp1435(A), Ala1431(A), Thr1279(A), Ala1283(A), Ile1427(A), Ser1430(A), Phe1464(A), Ser1467(A), Gly1439(A), Gly1440(A), Ala1441(A)
	NB‐598	−7.37	None	Asp1570(A), Ala1571(A), Ser1572(A), Glu400(A), Lys398(A), Ser399(A)
	Benzyl Sinapate (**6**)	−7.20	Ser1434(A) [6.54 Å], Arg1551(A) [6.54 Å]	Ile1427(A), Asp1435(A), Ser1430(A), Ala1431(A), Ser1467(A), Thr1279(A), Ala1283(A), Gly1439(A), Gly1440(A), Ala1441(A)
	4‐Chlorobenzyl Sinapate (**10**)	−7.11	Ser1434(A) [6.65 Å], Arg1551(A) [6.65 Å]	Ile1427(A), Asp1435(A), Thr1279(A), Ala1431(A), Phe1464(A), Ala1283(A), Ser1430(A), Gly1439(A), Gly1440(A), Ala1441(A), Ser1467(A)
	4‐Bromobenzyl Sinapate (**11**)	−6.94	Ser1434(A) [6.63 Å], Arg1551(A) [6.63 Å]	Ile1427(A), Asp1435(A), Ser1430(A), Ala1431(A), Phe1464(A), Ser1467(A), Thr1279(A), Ala1283(A), Gly1439(A), Gly1440(A), Ala1441(A)
	Hexyl Sinapate (**5**)	−6.74	Trp1528(A) [5.80 Å], Ser1462(A) [5.80 Å]	Asn1539(A), Gly1543(A), Tyr1544(A), Arg1546(A), Tyr1536(A), Arg1538(A), Ser1540(A), Met1547(A), Ser1564(A), Ser1567(A), Ala1568(A)
	Fluconazole	−6.72	Ser1567(A) [2.84 Å]	Met1547(A), Ile1459(A), Arg1538(A), Tyr1536(A), Asn1539(A), Gly1543(A), Tyr1544(A), Arg1546(A), Ala1568(A), Ser1462(A), Trp1528(A), Ser1564(A)
	Isopropyl Sinapate (**3**)	−6.49	Arg462(A) [2.86 Å], Ser1572(A) [2.86 Å], Lys398(A) [3.12 Å]	Gly1440(A), Gly1439(A), Ser399(A), Glu400(A), Ala1441(A)
	Methyl Sinapate (**1**)	−6.46	Arg462(A) [2.62 Å], His403(A) [2.62 Å], Gly1440(A) [5.93 Å], Ala1441(A) [5.93 Å], Asp402(A) [3.41 Å]	Gly1235(A), Gly1439(A), Lys398(A), Lys401(A), Ser1572(A)
	Propyl Sinapate (**2**)	−6.45	Ser1540(A) [6.67 Å]	Tyr1544(A), Met1547(A), Tyr1536(A), Gly1543(A), Ser1462(A), Arg1538(A), Ser1567(A)
	Pentyl Sinapate (**4**)	−6.03	Arg462(A) [4.15 Å], Lys398(A) [4.15 Å], Ser1572(A) [4.39 Å]	Gly1440(A), Glu400(A), Gly1439(A), Asp1570(A), Ala1441(A), Ala1571(A)

^a^ Binding energy of the best conformation obtained with AutoDock Vina v1.2.2 (exhaustiveness = 32). Ligand 3D structures were re‐generated from canonical SMILES validated against PubChem/ChEBI (OpenBabel –gen3d; Gasteiger charges).

^b^ Hydrogen‐bond donor–acceptor distances (Å) are reported in brackets. Residue lists correspond to the LigPlot+ analysis reported in the original manuscript; the qualitative interaction patterns remained consistent upon re‐docking with the corrected ligand structures.

### 2.6 Squalene Monooxygenase (PDB: 6C6P)

**FIGURE 1:**
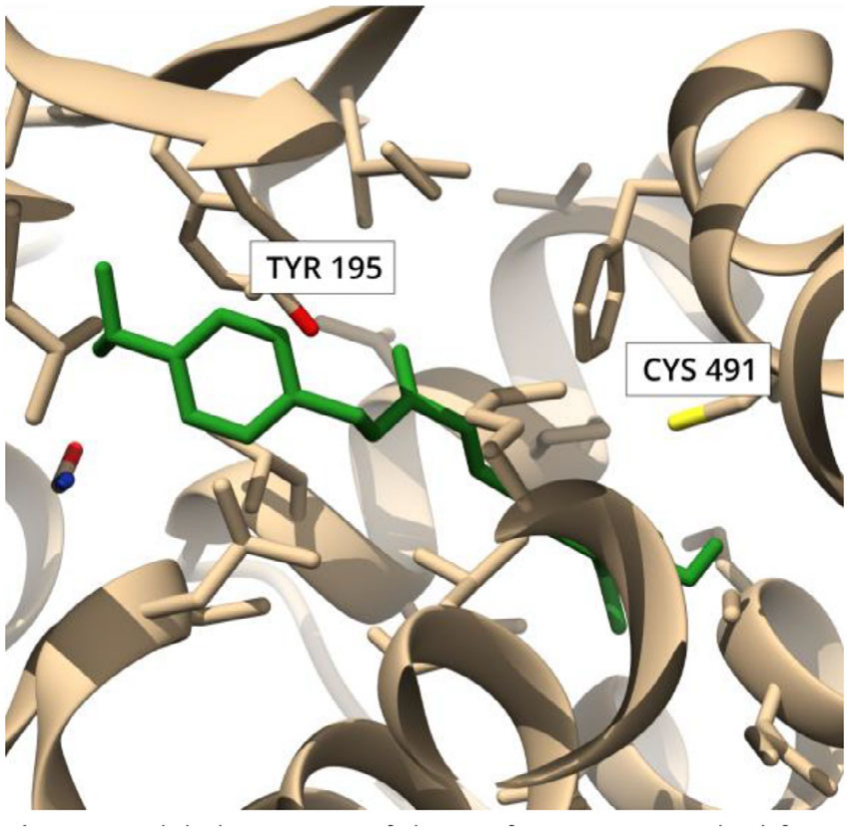
Global structure of the conformations resulted from docking. Representation in ribbons. Ligand: compound **8** (green) and Receptor: Squalene Monooxygenase (PDB: 6C6P).

Squalene monooxygenase exhibited the highest susceptibility to the sinapate derivatives among the targets analyzed. The cognate inhibitor NB‐598, co‐crystallized in 6C6P, displayed the highest predicted affinity (ΔGbind = −11.69 kcal/mol), as expected for a specific squalene epoxidase inhibitor. Among the synthesized sinapates, 4‐chlorobenzyl sinapate (**10**) and 4‐bromobenzyl sinapate (**11**) yielded the most favorable binding energies (−9.69 and −9.57 kcal/mol, respectively), followed closely by 4‐methylbenzyl sinapate (**7**, −9.37 kcal/mol) and benzyl sinapate (**6**, −8.83 kcal/mol). Tomudex (−9.58 kcal/mol) and fluconazole (−6.30 kcal/mol) were used as additional references; the lower score of fluconazole reinforces that squalene epoxidase is not its pharmacological target. Analysis of the binding mode revealed that the high‐affinity benzyl derivatives are stabilized by a conserved hydrogen bond with the Gln168(A) residue (Figure 1) and occupy a hydrophobic pocket composed of Phe166, Tyr195, Leu324, and Leu416, suggesting that para‐substituted benzyl groups optimize the van der Waals contacts within the catalytic site.

### 2.7 Lanosterol 14‐alpha‐Demethylase (PDB: 4WMZ)

**FIGURE 2:**
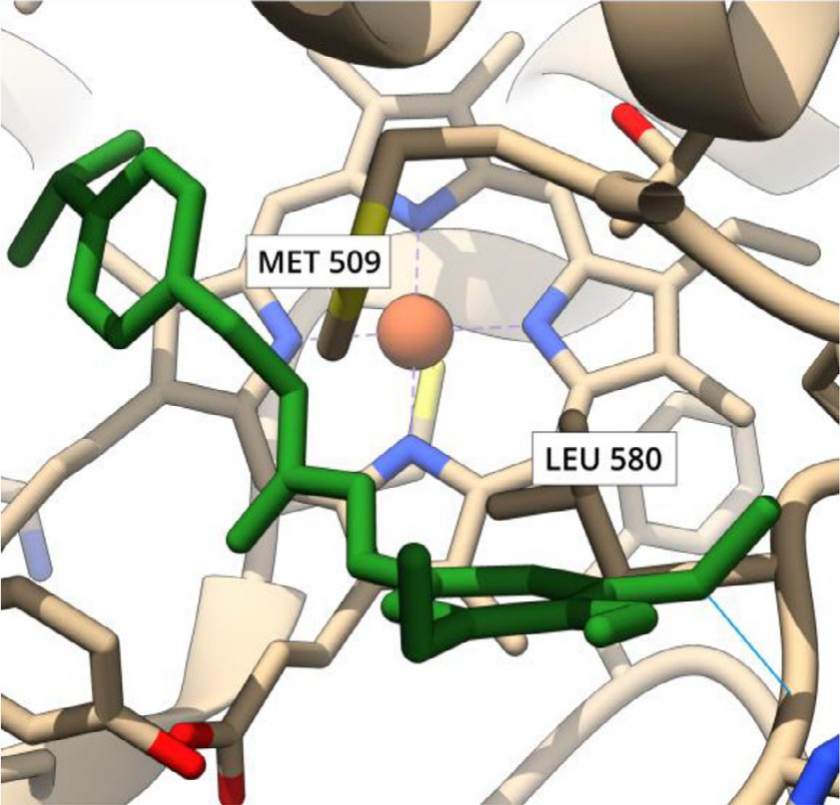
Global structure of the conformations resulted from docking. Representation in ribbons. Ligand: compound **8** (green) and Receptor: Lanosterol 14‐alpha‐Demethylase (PDB: 4WMZ).

For the 14α‐demethylase target, Tomudex (−9.75 kcal/mol) presented the highest affinity, closely followed by perilla sinapate (**9**, −9.72 kcal/mol), 4‐isopropylbenzyl sinapate (**8**, −9.32 kcal/mol), and 4‐methylbenzyl sinapate (**7**, −9.22 kcal/mol). Fluconazole exhibited a ΔGbind of −8.09 kcal/mol, comparable to the benzyl‐series sinapates (−8.57 to −8.94 kcal/mol). The differences observed between the top sinapates and fluconazole (0.5–1.6 kcal/mol) lie within the reported error margin of the AutoDock Vina scoring function (RMSE ≈ 2.85 kcal/mol), indicating comparable predicted affinities rather than statistically superior potency. A critical observation in this receptor was the interaction with the Hem601 prosthetic group. While alkyl derivatives like hexyl sinapate (−8.03 kcal/mol) formed direct interactions with the heme center, the benzyl derivatives were primarily stabilized by hydrogen bonds with Tyr140, Ser382 and Tyr126 (Figure 2), alongside an extensive hydrophobic network.

### 2.8 Delta(14)‐Sterol Reductase (PDB: 4QUV)

**FIGURE 3:**
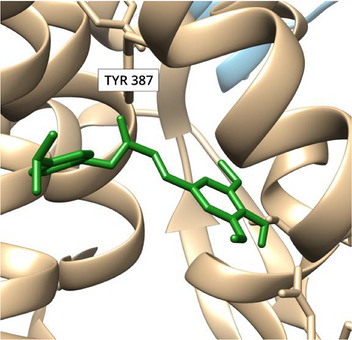
Global structure of the conformations resulted from docking. Representation in ribbons. Ligand: compound **8** (green) and Receptor: Delta(14)‐sterol Reductase (PDB: 4QUV).

The binding profiles for delta(14)‐sterol reductase were more moderate. Benzyl sinapate (**6**) emerged as the most effective sinapate in this group (−8.08 kcal/mol), narrowly followed by 4‐chlorobenzyl sinapate (**10**, −8.00 kcal/mol) and Tomudex (−7.99 kcal/mol). Fluconazole displayed a ΔGbind of −7.07 kcal/mol, again within the error margin of the scoring function relative to the top‐performing sinapates. Interestingly, the majority of the sinapate ligands, including the benzyl and alkyl series, demonstrated interactions with the Ndp501 (NADPH) cofactor. This suggests a mechanism of action centered on the disruption of the electron transfer required for sterol reduction, primarily through residues Arg395 and Asn359 (Figure 3).

### 2.9 Thymidylate Synthase (PDB: 6K7S)

**FIGURE 4:**
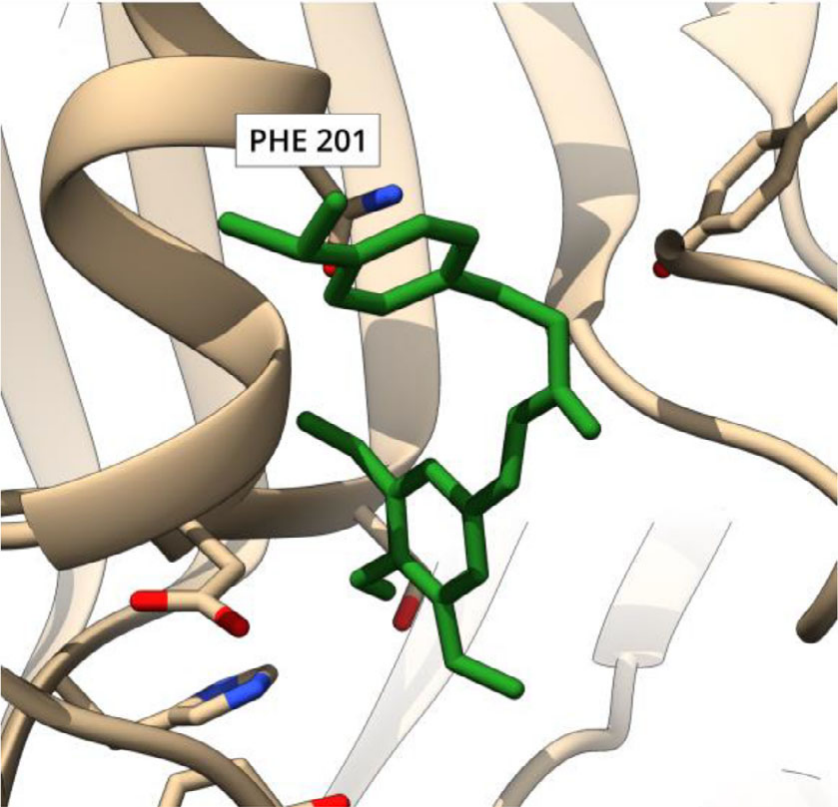
Global structure of the conformations resulted from docking. Representation in ribbons. Ligand: compound **8** (green) and Receptor: Thymidylate Synthase (PDB: 6K7S).

In thymidylate synthase simulations, Tomudex exhibited the highest stability (−8.17 kcal/mol), followed by 4‐isopropylbenzyl sinapate (**8**, −7.99 kcal/mol) and perilla sinapate (**9**, −7.81 kcal/mol); fluconazole presented −6.76 kcal/mol. Among the synthesized sinapates, the benzyl‐substituted derivatives outperformed the reference inhibitor NB‐598 (−7.62 kcal/mol), with 4‐isopropylbenzyl sinapate reaching −7.99 kcal/mol. The docking results highlighted a shift in the interaction pattern: while the benzyl series relied on hydrophobic interactions with Phe201 and Trp85, the shorter alkyl chains (e.g., methyl and propyl) favored polar interactions, forming hydrogen bonds with Cys171 and Asn202 (Figure 4). However, these polar contacts did not compensate for the loss of the hydrophobic surface area provided by the benzyl groups.

### 2.10 1,3‐Beta‐Glucan Synthase (GSC2 Component)

**FIGURE 5:**
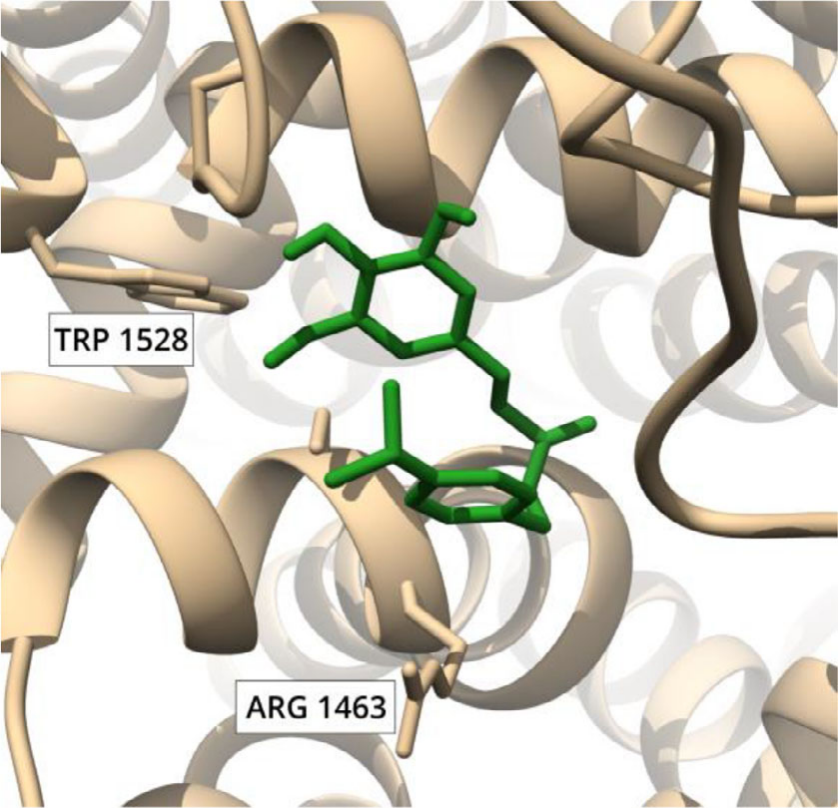
Global structure of the conformations resulted from docking. Representation in ribbons. Ligand: compound **8** (green) and Receptor: 1,3‐beta‐Glucan Synthase (GSC2 Component).

For the AlphaFold‐derived model of the GSC2 component, Tomudex (−8.32 kcal/mol) and perilla sinapate (**9**, −7.99 kcal/mol) were the most stable ligands, followed by 4‐isopropylbenzyl sinapate (**8**, −7.85 kcal/mol) and 4‐methylbenzyl sinapate (**7**, −7.43 kcal/mol). NB‐598 and fluconazole presented affinities of −7.37 and −6.72 kcal/mol, respectively. These ligands were consistently anchored by hydrogen bonds with Ser1434 and Arg1551 (Figure 5). The hydrophobic segment of the molecules was stabilized by a cluster including Ile1427, Phe1464, and Ala1283. In contrast, the alkyl derivatives showed a more fragmented binding pattern, with perilla and isopropyl sinapates interacting with different residues such as Arg462 and Ser1572.

Among the protein targets, Squalene Monooxygenase yielded the highest predicted affinities for the benzyl‐substituted sinapates, with 4‐chlorobenzyl sinapate (**10**, −9.69 kcal/mol), 4‐bromobenzyl sinapate (**11**, −9.57 kcal/mol), 4‐methylbenzyl sinapate (**7**, −9.37 kcal/mol), and benzyl sinapate (**6**, −8.83 kcal/mol) emerging as the most promising ligands of the series. The cognate inhibitor NB‐598 (−11.69 kcal/mol) confirmed the biological coherence of the docking protocol on this target, as expected for a specific squalene epoxidase inhibitor. These values reflect the high hydrophobic character of the active site of this protein, which favors aromatic side chains. Compound **8** (4‐isopropylbenzyl sinapate) also stood out across the CYP51‐family targets (4WMZ, 6K7S) and the GSC2 component of 1,3‐*β*‐glucan synthase, remaining competitive against Tomudex and fluconazole, with ΔGbind values within the error margin of the AutoDock Vina scoring function (RMSE ≈ 2.85 kcal/mol).

Although molecular docking results indicated higher binding affinities for benzyl‐substituted derivatives, this trend did not directly correlate with the in vitro antifungal activity, where alkyl‐chain derivatives, particularly compound **5**, exhibited superior potency. This discrepancy suggests that the antifungal mechanism is more likely associated with membrane interactions, as supported by the ergosterol assay, rather than specific enzyme inhibition. Therefore, while docking provides insights into possible molecular targets, lipophilicity and membrane affinity appear to be the dominant factors governing antifungal activity in this series. It is important to note that the ergosterol assay indicates that the antifungal activity of the most active compounds is associated with interactions at the fungal membrane. In this context, the molecular docking results are interpreted as complementary, suggesting possible interactions with enzymes involved in fungal metabolism, but not establishing a direct enzymatic mechanism of action. Therefore, the antifungal effect of the sinapate derivatives is likely related to membrane interaction, while the docking findings provide additional insight into potential molecular interactions.

## 3 Materials and Methods

### 3.1 Chemistry

The IR spectroscopy analysis was performed in the transformed Fourier spectrophotometer model IRPrestige‐21, from the manufacturer Shimadzu, present at LACOM‐Laboratory of Fuels and Materials‐UFPB. Nuclear Magnetic Resonance of Hydrogen (^1^H NMR) and Thirteen Carbon (^13^C NMR). The equipment used in the analyses was Mercury‐Varian spectrometers operating at 400 MHz (^1^H) and 100 MHz (^13^C), and Varian‐NMR‐System operating at 500 MHz (^1^H) and 125 MHz for (^13^C); available at the Multiuser Laboratory of Characterization and Analysis (LMCA/UFPB). In addition, the compounds unpublished in the literature were submitted to analysis by high‐resolution mass spectrometry with ionization by MALDI (Matrix assisted laser desorption/ionization).

### 3.2 Preparation of Sinapates 1–5

In a 250 mL flask, 0.4 g of sinapic acid (1.68 mmol) and 80 mL of the respective alcohol were added, then 0.8 mL of concentrated H_2_SO_4_ was slowly added. Thus, the reaction was conducted under reflux and magnetic stirring for 6 to 72 h. All reaction steps were monitored by CCDA [27, 28]. After the end of the reaction, the alcohol was partially removed under reduced pressure, then the product was transferred to a separation funnel and extraction was performed with 15 mL of distilled water and ethyl acetate (3×10 mL), separating the organic phase from the aqueous phase. The organic phase was then treated with 5% sodium bicarbonate (3×10 mL). Next, the organic phase was washed with 10 mL of distilled water and finally dried with anhydrous sodium sulfate (Na_2_SO_4_) and filtered the solution. After evaporation of the extracting liquid, the products obtained were purified by column chromatography using silica gel 60, and with hexane and ethyl acetate as mobile phase in increasing polarity gradient, a procedure accompanied by CCDA [27, 28].

*Hexyl sinapate* (**5**): Brown oil; Yield: 45.5% (249.5 mg); Reaction Time: 72 h; Retention Factor: 0.56 (7:3 Hex/AcOEt); IR ѵmax (KBr, cm^−1^): 3536; 3001; 2955; 2857; 1709; 1632; 1605; 1514; 1462; 1341; 1113; ^1^H NMR (CDCl_3_, 500 MHz): δ 7.59 (*d, J* = 15.9 Hz, H = 1); 6.77 (*s*, H = 2); 6.30 (*d*, *J* = 15.9 Hz, H = 1); 5.76 (*sl*, H = 1); 4.19 (*t*, *J* = 6.7 Hz, H = 2); 3.92 (*s*, H = 6); 1.70 (*quint*, *J =* 6.7, H = 2*); 1.42‐1.39 (m*, H = 2); 1.34‐1.32 (*m*, H = 4); 0.90 (*t*, *J* = 7 Hz, H = 3); ^13^C NMR (125 MHz, CDCl_3_): δ 167.41; 147.35; 144.96; 137.20; 126.13; 116.21; 105.17; 64.80; 56.48; 31.63; 28.88; 25.81; 22.70; 14.16; HRMS (MALDI) calculated for C_17_H_24_O_5_ [M + H]^+^: 309.1697, found 309.1692.

### 3.3 Preparation of Sinapates 6–9

In a 100 mL flask 0.1 g (0.42 mmol) of sinapic acid, 0.54 mmol of alcohol, 0.015 g of DMAP, and 6.3 mL of dichloromethane were added. After 5 min, 0.11 g of DCC (0.54 mmol) was added and the reaction was conducted under magnetic stirring and at room temperature for 24 to 48 h. At the end of the reaction, the solvent was partially removed under reduced pressure. The reaction product was filtered about three times to remove the DCU (poorly soluble in dichloromethane). The filtrate was transferred to a separatory funnel for extraction with 10 mL of distilled water and dichloromethane (3×10 mL). Then, the organic phase was treated with 10 mL of 5% sodium bicarbonate, followed by 10 mL of distilled water. The organic phase was dried with anhydrous sodium sulfate and filtered. The solvent was removed under reduced pressure, and the reaction product was purified in a silica gel 60 column with hexane and ethyl acetate mobile phase in an increasing polarity gradient. Purification and all stages of the reaction were monitored using CCDA [29, 30].

*4‐Methylbenzyl sinapate* (**7**): Yellow oil; Yield: 22.1% (32.5 mg); Reaction Time: 48 h; Retention Factor: 0.40 (7:3 Hex/AcOEt); IR ѵmax (KBr, cm^−1^): 3437; 3009; 2980; 2846; 1696; 1630; 1600; 1512; 1453;; 1338; 1109; ^1^H NMR (CDCl_3_, 400 MHz): δ 7.62 (*d*, *J* = 15.9 Hz, H = 1); 7.31 (*d*, *J* = 8.1 Hz, H = 2); 7.19 (*d*, *J* = 7.9 Hz, H = 2); 6.76 (*s*, H = 2); 6.34 (*d, J* = 15.9 Hz, H = 1); 5.75 (*s*, H = 1); 5.20 (*s*, H = 2); 3.91 (*s*, H = 6); 2.36 (*s*, H = 3); ^13^C NMR (100 MHz CDCl_3_): δ 167.12; 147.34; 145.42; 138.28; 137.28; 133.25; 129.43, 128.62; 126.06; 115.93; 105.20; 66.42; 56.47; 21.36; HRMS (MALDI) calculated for C_19_H_20_O_5_ [M + H] ^+^: 329.1384, found 329.1382

*4‐Isopropylbenzyl sinapate* (**8**): White oil; Yield: 26.8% (42.6 mg); Reaction Time: 48 h; Retention Factor: 0.44 (7:3 Hex/AcOEt); IR ѵmax (KBr, cm^−1^): 3323; 2955; 2851; 1713; 1626; 1593; 1514; 1452; 1310; 1113; ^1^H NMR (CDCl_3_, 400 MHz): δ 7.73 (*d*, *J* = 15.9 Hz, H = 1); 7.45 (*d*, *J* = 8.3 Hz, H = 2); 7.35 (*d*, *J* = 8.6 Hz, H = 2); 6.86 (*s*, H = 2); 6.45 (*d*, *J* = 15.9 H = 1); 5.89 (*s*, H = 1); 5.31 (*s*, H = 2); 4.01 (*s*, H = 6); 3.03 (*sept*, *J* = 6.9, H = 1); 1.36 (*d, J* = 8.0 Hz, H = 6); ^13^C NMR (100 MHz CDCl_3_): δ 167.14; 149.26; 147.35; 145.43; 137.29; 133.61; 128.68; 126.84; 126.06; 115.93; 105.21; 66.43; 56.47; 34.06; 24.11; HRMS (MALDI) calculated for C_21_H_21_O_5_ [M + H]^+^: 357.1697, found 357.1696.

*Perilla sinapate* (**9**): White oil; Yield: 13.9% (22.3 mg); Reaction Time: 48 h; Retention Factor: 0.46 (7:3 Hex/AcOEt); IR ѵmax (KBr, cm^−1^): 3420; 3080; 3005; 2849; 1708; 1632; 1599; 1514; 1452; 1339; 1113; ^1^H NMR (CDCl_3_, 400 MHz): δ 7.60 (*d*, *J* = 15.9 Hz, H = 1); 6.77 (*s*, H = 2); 6.32 (*d, J* = 15.9 Hz, H = 1); 5.82 (*sl*, H = 1); 5.76 (*s*, H = 1); 5.29 – 4.72 (*m*, H = 2); 4.59 (*s*, H = 2); 3.92 (*s*, H = 6); 2.19–2.14 (*m*, H = 4); 2.04–1.96 (*m*, H = 1); 1.89–1.86 (*m*, H = 1); 1.74 (*s*, H = 3); 1.52 (*m*, H = 1); ^13^C NMR (100 MHz CDCl3): δ 167.20; 149.77; 147.35; 145.21; 137.26; 132.93; 126.09; 125.97; 116.03; 108.94; 105.19; 68.57; 56.49; 41.01; 30.65; 27.50; 26.63; 20.90; HRMS (MALDI) calculated for C_21_H_26_O_5_ [M + H]^+^: 359.1853, found 359.1850.

### 3.4 Preparation of Sinapates 10–11

In a 100 mL flask 0.1 g (0.42 mmol) of sinapic acid and 0.43 mmol of the corresponding benzyl halide were added, dissolving in 5 mL of anhydrous acetone in the presence of 1.68 mmol of triethylamine. The reaction occurred for 48 h and was conducted under reflux and magnetic agitation. Then, the acetone was removed under reduced pressure, and the product was extracted with 10 mL of distilled water and dichloromethane (3×10 mL). The organic phase obtained was treated with 5% NaHCO_3_ (3×10 mL), washed with 10 mL of distilled water, and dried with anhydrous sodium sulfate. After extraction, the solvent was evaporated under reduced pressure and the product was purified by column chromatography using silica gel 60 and a mixture of hexane and ethyl acetate with increasing polarity gradient [31, 32].

*4‐Chlorobenzyl sinapate (* ***10*** *)*: Yellow crystalline solid; Yield: 57.6% (374.3 mg); Melting Point: 134.4°C; Reaction Time: 48 h; Retention Factor: 0.38 (7:3 Hex/AcOEt); IR ѵmax (KBr, cm^−1^): 3510; 3009; 2959; 2849; 1704; 1638; 1610; 1516; 1454; 1344; 1115; ^1^H NMR (CDCl_3_, 400 MHz): δ 7.62 (*d, J = 15.9 Hz, H = 1); 7.35 (m*, H = 4); 6.76 (*s*, H = 2); 6.33 (*d, J* = 15.9 Hz, H = 1); 5.20 (*s*, H = 2); 3.91 (*s*, H = 6); ^13^C NMR (100 MHz CDCl_3_): δ 166.98; 147.36; 145.85; 137.43; 134.78; 134.26; 129.78; 128.90; 125.88; 115.44; 105.27; 65.57; 56.47; HRMS (MALDI) calculated for C_18_H_17_ClO_5_ [M + H]^+^: 349.0837, found 349.0829.

*4‐Bromobenzyl sinapate* (**11**): Yellow crystalline solid; Yield: 49.4% (330.2 mg); Melting Point: 132.5°C; Reaction Time: 48 h; Retention Factor: 0.38 (7:3 Hex/AcOEt); IR ѵmax (KBr, cm^−1^): 3496; 3007; 2968; 2849; 1703; 1639; 1609; 1514; 1454; 1342; 1115; ^1^H NMR (CDCl_3_, 500 MHz): δ 7.62 (*d, J = 15.9 Hz, H = 1); 7.50 (d, J* = 8.4, H = 2); 7.28 (*d, J* = 8.4, H = 2); 6.76 (*s*, H = 2); 6.33 (*d, J* = 15.9 Hz, H = 1); 5.18 (*s*, H = 2); 3.91 (*s*, H = 6); ^13^C NMR (125 MHz CDCl_3_): δ 166.94; 147.36; 145.86; 137.43; 135.31; 131.87; 130.06; 125.89; 122.40; 115.43; 105.27; 65.59; 56.44; HRMS (MALDI) calculated for C_18_H_17_BrO_5_ [M + H]^+^: 393.0332, found 393.0336.

### 3.5 In Vitro Antifungal Activity Assay Against Candida spp. Strains

The yeast suspension was prepared in RPMI broth (Roswell Park Memorial Institute medium, Sigma, Darmstadt, Germany) and adjusted with turbidity equivalent to 2.5 10^3^ CFU/mL, 530 nm, abs 0.08–0.13, using strains of *C. albicans* ATCC 60193 and *C. tropicalis* ATCC 750 [33, 34]. Initially, a screening was performed with the eleven sinapates (**1‐11**), at a maximum concentration of 1000 µg/ml, and their activity was later evaluated against *Candida* spp. strains. Sterilized microdilution plates with 96 flat‐bottomed wells initially received 100 µL of RPMI. Then, 100 µL of the test substances were inserted into the first well of each column, followed by the serial microdilution process, providing concentrations between 1000 and 7.81 µg/ml. Subsequently, 100 µL of the inoculum of the fungal strains was added to each well, and the positive control, nystatin, at concentrations that alternated from 48 to 0.75 µg/ml. The plates were incubated for 24 h at 35°C, and the results were read by visual observation. The MIC was defined as the lowest concentration capable of inhibiting visible fungal growth, as determined by visual inspection, and was expressed as the most frequently observed value (mode) across three independent experiments, each performed in triplicate.

The antifungal activity of the three esters was interpreted according to the following criteria: (a) very strong bioactivity (MIC <3.515 µg/ml); (b) strong bioactivity (MIC between 3.515 and 25 µg/ml); (c) moderate bioactivity (MIC between 26 ‐100 µg/ml); (d) weak bioactivity (MIC from 101 to 500 µg/ml); (e) very weak bioactivity (MIC in the range of 501‐2000 µg/ml) [34, 35, 36]. MFC was determined after reading the MIC, aliquots of 20 µL corresponding to the MIC, and two multiple concentrations of MIC were subcultured on Sabouraud Dextrose agar (KASVI1, Kasv Imp and Dist de Prod/Laboratorios LTDA, Curitiba, Brazil). These plates were incubated for 24 h at 35°C. The reading of the results consisted of visual observation of the fungal growth in the culture medium. Next, the MFC/MIC ratio was calculated to determine whether the substance was fungistatic (MFC/MIC ≥ 4) or fungicidal (MFC/MIC <4) [37, 38].

### 3.6 Sorbitol Assay

The test was performed by determining the MIC in the presence and absence of sorbitol 0.8 M (anhydrous D‐sorbitol), to compare the MIC values of substances 4,5, and 8 against the *C. albicans* strain *ATCC 60193*. Caspofungin was used as a positive control at an initial concentration of 16 to 0.125 µg/mL. The increase in the MIC value in the presence of sorbitol will indicate the cell wall as one of the possible targets of the test substance [39, 40, 41]. The experiments were performed in triplicate in three independent experiments.

### 3.7 Ergosterol Trial

The methodology used in this step is like microdilution, differing in the culture medium in which exogenous ergosterol (Sigma‐Aldrich, São Paulo, Brazil) will be added at a concentration of 400 µg/ml. Some antifungal agents act on plasma membrane ergosterol by forming complexes or inhibiting membrane biosynthesis. Thus, the increase in MIC in the presence of exogenous ergosterol will indicate the activity of the agent under study on the fungal plasma membrane [41]. Nystatin was used as a positive control, due to its activity on the plasma membrane. The experiments were performed in triplicate and three independent experiments.

### 3.8 Analysis of Antifungal Susceptibility Data

All experiments were performed in triplicate and repeated independently three times (n = 9). MIC and MFC values were determined based on the visual assessment of fungal growth inhibition. As these endpoints correspond to discrete concentration values associated with the presence or absence of visible growth, rather than continuous quantitative variables, results were expressed as the mode of the nine determinations. Because complete or near‐complete agreement was observed among replicates, measures of variability and inferential statistical analyses were not considered appropriate and were therefore not applied. Descriptive statistical analysis was performed using Jamovi software (version 2.6.26).

### 3.9 ADMET Predictions Methodology

The 3D structures of ligands and SMILES code were obtained from the PubChem platform [42] (compounds **1** to **4**) and Avogadro software [43] version 1.2.0 (https://avogadro.cc/) (compounds **5** to **11**). Toxicity, water solubility, physicochemical, and pharmacokinetic properties of the ligands were obtained from online platforms: In Silico Toxicology [44] (https://lazar.in‐silico.ch/predict), ProTox‐II—Prediction of Toxicity of Chemicals [45] (https://tox‐new.charite.de/protox_II/), and SwissADME [46] (http://www.swissadme.ch/). SWISSADME [47] estimates the main parameters used in research, such as Lipinski's rule (rule of five), water solubility (LogS), pharmacokinetic parameters such as the potential to cross the blood‐brain barrier (BBB), Human Intestinal Absorption Capacity (HIA), and P‐glycoprotein (P‐gp). ProTox‐II [48] predicts the toxicological profile of compounds based on mutagenicity (*Salmonella typhimurium*), carcinogenicity (in rats and rodents), maximum recommended daily dose (MRDD), and other parameters.

### 3.10 Molecular Docking Methodology

The three‐dimensional (3D) structures of squalene monooxygenase, lanosterol 14‐alpha‐demethylase, delta (14)‐sterol reductase, and thymidylate synthase were retrieved from the Protein Data Bank (PDB) [47] under the accession codes 6C6P [48, 49], 4WMZ [50, 51], 4QUV [52, 53], and 6K7S [54, 55], respectively. Due to the absence of an experimental structure, the 3D model for the GSC2 component of 1,3‐beta‐glucan synthase was obtained from the AlphaFold Protein Structure Database [56] (UniProt [57] entry P40989). Pre‐processing of the receptor structures involved the removal of co‐crystallized ligands and noncatalytic molecules (e.g., water molecules and ions) not involved in the binding site. Subsequently, the protein structures were prepared and protonated using PDB2PQR [58], with pKa values and protonation states assigned by PROPKA [59] at physiological pH. Final structural refinements and file conversions were performed using AutoDockTools 4 [60].

### 3.11 Ligand Selection and Preparation

The 3D structures of the ligands Tomudex (PubChem CID 135400182), NB‐598 (PubChem CID 6443223), and Fluconazole (PubChem CID 3365) were extracted directly from their respective PDB co‐crystal complexes (6K7S, 6C6P, and 4WMZ). All ligands were prepared for docking simulations—including the assignment of Gasteiger charges and rotatable bonds—using AutoDockTools 4.

### 3.12 Docking Parameters and Execution

Molecular docking simulations were executed using AutoDock Vina (v1.2.2) [61]. To ensure an extensive search of the conformational space, the calculations were performed with an exhaustiveness value of 32; all other parameters were maintained at their default settings [62, 63]. The specific coordinates for the grid center and the dimensions of the search space (grid box) for each receptor are detailed in Table 7.

**TABLE 7:** Grid Configuration Parameters for Autodock Vina Molecular Docking Simulations.

Proteins	Parameters of the grid	Exhaustiveness
Squalene monooxygenase (PDB ID: 6C6P [41, 42] chain B)	Center: (−20.36, 78.16, 57.63). Size (in angstroms): X 24 Y 24 Z 24	32
Lanosterol 14α‐Demethylase (PDB ID: 4WMZ [43, 44])	Center: (22.92, 10.43, 16.03). Size (in angstroms): X 24 Y 24 Z 24	32
Δ(14)‐sterol reductase (PDB ID: 4QUV [45, 46])	Center: (−36.21, −2.61, 15.58). Size (in angstroms): X 24 Y 24 Z 24	32
Thymidylate synthase (PDB ID: 6K7S [47, 48] chain A)	Center: (46.14, −6.11, 18.62). Size (in angstroms): X 24 Y 24 Z 24	32
1,3‐β‐glucan synthase (AlphaFold [50, 57] model, Uniprot P40989)	Center: (−3.00, 18.00, −4.00). Size (in angstroms): X 24 Y 24 Z 24	32

### 3.13 Validation of the Molecular Docking Protocol

To ensure the reliability of the docking protocol employed, we performed validation through the redocking of co‐crystallized ligands into their native crystal structures. Receptors 6c6p (Squalene Epoxidase) and 4wmz (Lanosterol 14*α*‐Demethylase, CYP51) were co‐crystallized with NB‐598 (PDB ligand code: EMV) and fluconazole (PDB ligand code: TPF), respectively [64]. Redocking validation was performed for the receptors co‐crystallized with well‐resolved small‐molecule inhibitors (6c6p and 4wmz); the remaining structures were co‐crystallized with cofactors or partially resolved ligands unsuitable as redocking references.

Receptors were prepared using *prepare_receptor* (ADFR Suite): crystallographic water molecules were removed, polar hydrogen atoms were added, and Gasteiger partial charges were assigned. Essential cofactors required for active site integrity were retained — flavin adenine dinucleotide (FAD) for 6c6p and the iron‐protoporphyrin IX (heme) group for 4wmz — and QEq charges were assigned to the heme Fe center to preserve appropriate electrostatic representation. Ligands were converted from SDF to PDBQT format using *mk_prepare_ligand.py* (ADFR Suite), with Gasteiger charges assigned and rotatable bonds detected automatically. Receptors were treated as rigid bodies, while all rotatable bonds of the ligands were kept flexible.

The redocking protocol employed the same grid box parameters and exhaustiveness value (32) used in the primary docking studies. The grid box center was defined at the geometric centroid of the co‐crystallized ligand heavy‐atom coordinates in each receptor (6c6p: −20.36, 78.16, 57.63; 4wmz: 22.92, 10.43, 16.03), with dimensions of 24 × 24 × 24 Å and spacing of 0.375 Å. The grid dimension was selected following the criterion of approximately 2.9 times the ligand radius of gyration, as recommended by [65] for the optimization of binding affinity predictions. Nine binding modes (*num_modes* = 9) were generated per run using AutoDock Vina v1.2.2 [61], with calculations parallelized across 8 CPU cores.

The predicted binding poses were compared against the crystallographic structures, and the root‐mean‐square deviation (RMSD) was calculated over all heavy atoms of the ligands using obrms (Open Babel v3.1.1) [66], which employs graph isomorphism‐based atom matching to correctly handle chemically equivalent atoms (e.g., symmetric phenyl substituents). No structural alignment was applied, as both crystallographic and predicted poses share the receptor coordinate frame. The lowest‐energy pose (mode 1) was taken as the predicted binding mode. RMSD values < 2.0 Å are widely accepted as indicative of a reliable protocol for binding mode prediction [67].

The redocking yielded RMSD values of 0.55 Å for NB‐598 in 6c6p and 1.54 Å for fluconazole in 4wmz (Table 1), both below the 2.0 Å acceptance threshold, confirming the reliability of the protocol. For 4wmz, the Fe–N coordination bond between the triazole ring of fluconazole and the heme iron is not explicitly modeled by the Vina empirical scoring function; nevertheless, the protocol successfully reproduced the crystallographic binding geometry within the accepted threshold.

## 4 Conclusion

The hexyl sinapate (**5**) showed higher antifungal potency with MIC and MFC equal to 25.33 µM against *C. albicans* and *C. tropicalis*. Compound **8** also showed strong bioactivity against C. albicans strains with MIC and MFC equal to 43.83 µM. In the molecular docking analysis, this compound exhibited competitive binding energies across the selected protein targets, particularly against the CYP51‐family enzymes and the GSC2 component of 1,3‐*β*‐glucan synthase, while the benzyl series— especially compounds **6**, **7**, **10**, and **11** — displayed the highest predicted affinities against squalene monooxygenase. Taken together, the docking and ergosterol‐assay results support the fungal plasma membrane as the most likely target for the antifungal activity of this series. Meanwhile, compound **4** showed strong bioactivity against *C. tropicalis* strains, with MIC of 53.43 µM. The analysis of the structure‐activity relationship indicated that the increase in the carbon chain potentiated the antifungal activity. The presence of the benzyl group in compound **6**, as well as in the benzyl derivatives with substituents at the *para* position, **7, 10,** and **11,** showed MIC and MFC at higher concentrations, which did not occur with compound **8**, which has an isopropyl group at the *para* position of the aromatic ring. The trials of ergosterol and sorbitol with compounds **4**, **5**, and **8** showed that the compounds act at the plasma membrane level and have no effect on the cell wall. The ADMET study predicted that the compounds have good gastrointestinal absorption, absence of toxicity in rats and mice, and a low lethal dose.

## Author Contribution Statement

The study was conducted, the data were organized, and the original manuscript was written by **F. E. d. S. F**. and j**. J. E**. the tests to evaluate antifungal activity were carried out by p**. L. N**. and r**. D. d. C**. the silico study was developed by é**. R. A. d. S., R**. o**. B**., and r**. M. R**. the project's conception and coordination were led by d**. P. d. S**. All authors reviewed the manuscript's content and approved the final version for publication.

## Funding Statement

This work received financial support from Brazilian government agencies National Council for Scientific and Technological Development (CNPq) and Coordination for the Improvement of Higher Education Personnel (CAPES).

## Conflicts of Interest

The authors declare no conflict of interest.

## Supporting Information

The following Supporting Information can be downloaded: Figures 1–39: ^1^H and ^1^ ^3^C NMR, infrared spectroscopy, and HRMS spectra of compounds **1**‐**11**.

## Supporting information




**Supporting File 1**: cbdv71569‐sup‐0001‐SuppMat.docx.

## Data Availability

The data that support the findings of this study are openly available in Universidade Federal da Paraíba at https://repositorio.ufpb.br/jspui/bitstream/123456789/26283/1/FernandoEmanuelDeSousaFerreira_Dissert.pdf.
