# Supplementary material for: Synthetic Sinapates: Antifungal, Molecular Docking, and ADMET Studies
Source: Chem Biodivers. 2026 Aug 9;23(8):e71569. doi: 10.1002/cbdv.71569 (PMC13453077; doi:10.1002/cbdv.71569)
Supplement: Supplementary file 1 [file CBDV-23-e71569-s001.docx]

**Supporting Information**

**Synthetic Sinapates: Antifungal, Molecular Docking, and ADMET Studies**

Fernando Emanuel de Sousa Ferreira^[a],^ Jeremias Justo Emídio^[a]^, Ézio Raul Alves de Sá^[b]^, Rômulo Oliveira Barros^[b]^, Paula Lima Nogueira^[c]^, Ricardo Martins Ramos^[b,d]^, Ricardo Dias de Castro^[c]^, Damião Pergentino de Sousa^[a],^*

^[a]^Laboratory of Pharmaceutical Chemistry, Department of Pharmaceutical Sciences, Federal University of Paraíba, João Pessoa 58051-900, Paraíba, Brazil;

^[b]^LaPeSI–Information Systems Research Laboratory, Department of Information, Environment, Health and Food Production, Federal Institute of Piauí, Teresina, Piauí, Brazil;

^[c]^Laboratory of Experimental Pharmacology and Cell Culture, Department of Clinical and Social Dentistry, Federal University of Paraiba, João Pessoa, Paraíba, Brazil;

^[d]^LaBME–Laboratory of Molecular Biology and Epidemiology, Federal Institute of Education,

Science and Technology of Piauı́–Campus Teresina Central, Teresina, Piauí, Brazil;

*Corresponding author: dps2@academico.ufpb.br

**Chemical characterization of compounds 1-11**

**Methyl sinapate (1):** Yellow crystalline solid; Yield: 72.7% (306.6 mg); Melting point: 87.7ºC; Reaction time: 6 hours; Retention factor: 0.32 (7:3 Hex/AcOEt); ); **IR ʋmax (KBr, cm^-1^):** 3005; 2986; 2846; 3524; 1696; 1603; 1425; 1514; 1461; 826; 1333; 1101; **^1^H NMR (CDCl_3_, 400 MHz):** δ 7.59 (*d, J*=15.9 Hz, H=1); 6.76 (*s*, H=2); 6.29 (*d, J* = 15,9 Hz, H=1); 5.81 (*s*, H=1); 3.91 (*s*, H=6); 3.79 (*s*, 3H); **^13^C NMR (CDCl_3_,** 100 MHz): δ 167.15; 147.33; 145.29; 137.29; 125.99; 115.67; 105.15; 56.57; 51.75.

**Figure 1:** Infrared spectrum ʋmax (KBr, cm^-1^) of compound **1**

**Figure 2**: ^1^H NMR spectrum (400 MHz, CDCl_3_) of compound **1**

**Figure 3:** ^13^C NMR spectrum (APT, 100 MHz, CDCl_3_) of compound **1**

**Propyl sinapate (2):** Yellow crystalline solid; Yield: 82.3% (388.3 mg); Melting point: 75.8ºC; Reaction time: 6 hours; Retention factor: 0.52 (7:3 Hex/AcOEt); **IR ʋmax (KBr, cm^-1^):** 3007; 2967; 2843; 3524; 1678; 1631; 1454; 1585; 1511; 821; 1332; 1107; **^1^H NMR (CDCl_3_, 400 MHz):** δ 7.59 (*d, J*=15.9 Hz, H=1); 6.77 (*s*, H=2); 6.31 (*d, J* = 15.9 Hz, H=1); 5.79 (*s*, H=1); 4.16 (*t, J* = 6,7 Hz, H=2); 3.91 (*s*, H=6); 1.72 (*sext*, *J*= 7.2 Hz H=2); 0.99 (*t*, *J* = 7,4 Hz, H=3); **^13^C NMR (CDCl_3_,** 125 MHz): δ 167.30; 147.21; 144.87; 137.07; 125.97; 116.03; 105.03; 66.09; 56.34; 22;13; 10.48.

**Figure 4**: Infrared spectrum ʋmax (KBr, cm^-1^) of compound **2**

**Figure 5**: ^1^H NMR spectrum (400 MHz, CDCl_3_) of compound **2**

**Figure** 6: ^13^C NMR spectrum (KBr, 125 MHz, CDCl_3_) of compound **2**

**Isopropyl sinapate (3):** Brown amorphous solid; Yield: 78.1% (368.5 mg); Melting point: 70.6ºC; Reaction time: 6 hours; Retention factor: 0.52 (7:3 Hex/AcOEt); **IR ʋmax (KBr, cm^-1^):** 3019; 2976; 2872; 3552; 1682; 1631; 1454; 1599; 1513; 816; 1332; 1107; **^1^H NMR (CDCl_3_, 400 MHz):** δ 7.56 (*d*, *J*=15.9 Hz, H=1); 6.76 (*s*, H=2); 6.27 (*d, J* = 15.9 Hz, H=1); 5.79 (*s*, H=1); 5.13 (*sept*, *J* = 6.3 Hz, H=1); 3.90 (*s*, H=6); 1.30 (*d, J* = 6,2 Hz, 6H; **^13^C NMR (CDCl_3_,** 100 MHz): δ 166.65; 147.19; 144.57; 136.99; 126.00; 116.55; 104.97; 67.64; 56.30; 21.96.

**Figure 7**: Infrared spectrum ʋmax (KBr, cm^-1^) of compound 3

**Figure 8**: ^1^H NMR spectrum (400 MHz, CDCl_3_) of compound **3**

**Figure** 9: ^13^C NMR spectrum (KBr, 125 MHz, CDCl_3_) of compound **3**

**Pentyl sinapate (4):** Brown oil; Yield; 33.6% (175.4 mg); Reaction time: 72 hours; Retention factor: 0.56 (7:3 Hex/AcOEt); **IR ʋmax (KBr, cm^-1^):** 3005; 296; 2868; 3572; 1690; 1634; 1454; 1585; 1514; 825; 1279; 1113; **^1^H NMR (CDCl_3_, 500 MHz):** δ 7.58 (*d*, *J=*15.9 Hz, H=1); 6.76 (*s*, H=2); 6.30 (*d*, *J* = 15.9 Hz, H=1); 5.82 (*s*, H=1); 4.18 (*t*, *J* = 6.80 Hz, H=2); 3.91 (*s*, H=6); 1.73-1.67 (*m*, H=2); 1.39 – 1.35 (*m*, H=4); 0.91 (*t,J* = 7.20 Hz, H=3) **^13^C NMR (CDCl_3_,** 125 MHz): δ 167.35; 147.22; 144.89; 137.10; 125.96; 116.01; 106.06; 64.67; 56.34; 28.47; 28.14; 22.37; 13.98.

**Figure 10:** Infrared spectrum ʋmax (KBr, cm^-1^) of compound **4**

Figure11: ^1^H NMR spectrum (500 MHz, CDCl_3_) of comp 4

**Figure 12:** ^13^C NMR spectrum (KBr, 125 MHz, CDCl_3_) of compound 4

**Hexyl sinapate (5):**

**Figure 13**: Infrared spectrum ʋmax (KBr, cm^-1^) of compound **5**

**Figure 14:** ^1^H NMR spectrum (500 MHz, CDCl_3_) of compound 5

**Figure 15:** ^13^C NMR spectrum (KBr, 125 MHz, CDCl_3_) of compound **5**

**Figure 16**: HRMS spectrum of compound **5**

**Benzyl sinapato (6):** Yellow oil; Yield: 32.4% (45.5 mg); Reaction time: 48 hours; Retention factor; 0.44 (7:3 Hex/AcOEt); **IR ʋmax (KBr, cm^-1^):** 3009; 2995; 285; 3499; 1703; 1639; 1452; 1612; 1515; 842; 1289; 1120; **^1^H NMR (CDCl_3_, 500 MHz):** δ 7,63 (*d*, *J* =15.9 Hz, H=1); 7.43 – 7.35 (*m*, H=5); 6.77 (*s*, H=2); 6.35 (*d*, *J* = 15.9 Hz, H=1); 5.78 (*s*, H=1); 5.24 (*s*, H=2); 3.91 (*s*, H=6); **^13^C NMR (CDCl_3_,** 125 MHz): δ 167.07; 147.35; 145.56; 137.33; 136.27; 128.74; 128.43; 128.39; 126.01; 115.77; 105.23; 66.44; 56.47.

**Figure 17:** Infrared spectrum ʋmax (KBr, cm^-1^) of compound **6**

**Figure 18:** ^1^H NMR spectrum (500 MHz, CDCl_3_) of compound **6**

**Figure 19:** ^13^C NMR spectrum (KBr, 125 MHz, CDCl_3_) of compound **6**

**4-Methylbenzyl sinapate (7):**

**Figure 20:** Infrared spectrum ʋmax (KBr, cm^-1^) of compound **7**

**Figure21:** ^1^H NMR spectrum (400 MHz, CDCl_3_) of compound **7**

**Figure 22:** ^13^C NMR spectrum (KBr, 125 MHz, CDCl_3_) of compound **7**

**Figure23**: HRMS spectrum of compound **7**

**4-isopropylbenzyl shoe (8):**

**Figure 24:** Infrared spectrum ʋmax (KBr, cm^-1^) of compound **8**

**Figure 25:** ^1^H NMR spectrum (400 MHz, CDCl_3_) of compound **8**

**Figure 26**: ^13^C NMR spectrum (KBr, 125 MHz, CDCl_3_) of compound **8**

**Figure 27**: HRMS spectrum of compound **8**

**Perilla sinapate (9):**

**Figura 28:** Infrared spectrum ʋmax (KBr, cm^-1^) of compound **9**

**Figure 29**: ^13^C NMR spectrum (KBr, 100 MHz, CDCl_3_) of compound **9**

**Figure 30:** ^1^H NMR spectrum (400 MHz, CDCl_3_) of compound **9**

**Figure 31**: HRMS spectrum of compound **9**

**4-Chlorobenzyl sinapate (10):**

**Figure 32:** Infrared spectrum ʋmax (KBr, cm^-1^) of compound **10**

**Figure 33:** ^1^H NMR spectrum (400 MHz, CDCl_3_) of compound **10**

**Figure 34:** ^13^C NMR spectrum (KBr, 100 MHz, CDCl_3_) of compound **10**

**4-Bromobenzyl sinapate (11)****:**

**Figure 35:**  HRMS spectrum of compound **10**

**Figure 36:** Infrared spectrum ʋmax (KBr, cm^-1^) of compound **11**

**Figure 37:** ^1^H NMR spectrum (400 MHz, CDCl_3_) of compound **11**

**Figure 38:** ^13^C NMR spectrum (KBr, 100 MHz, CDCl_3_) of compound **11**

**Figure 39**: HRMS spectrum of compound **11**
